# A high‐fat diet exacerbates the Alzheimer's disease pathology in the hippocampus of the *App* *^NL−F/NL−F^* knock‐in mouse model

**DOI:** 10.1111/acel.13429

**Published:** 2021-07-10

**Authors:** Guianfranco Mazzei, Ryohei Ikegami, Nona Abolhassani, Naoki Haruyama, Kunihiko Sakumi, Takashi Saito, Takaomi C. Saido, Yusaku Nakabeppu

**Affiliations:** ^1^ Division of Neurofunctional Genomics Department of Immunobiology and Neuroscience Medical Institute of Bioregulation Kyushu University Fukuoka Japan; ^2^ Laboratory for Proteolytic Neuroscience RIKEN Center for Brain Science Saitama Japan; ^3^ Department of Neurocognitive Science Institute of Brain Science Nagoya City University Graduate School of Medical Sciences Nagoya Japan

**Keywords:** Alzheimer's disease, β amyloid, gene expression, knock‐in mouse model, microgliosis, oxidative stress, transthyretin, type 2 diabetes mellitus

## Abstract

Insulin resistance and diabetes mellitus are major risk factors for Alzheimer's disease (AD), and studies with transgenic mouse models of AD have provided supportive evidence with some controversies. To overcome potential artifacts derived from transgenes, we used a knock‐in mouse model, *App^NL−F/NL−F^*, which accumulates Aβ plaques from 6 months of age and shows mild cognitive impairment at 18 months of age, without the overproduction of APP. In the present study, 6‐month‐old male *App^NL−F/NL−F^* and wild‐type mice were fed a regular or high‐fat diet (HFD) for 12 months. HFD treatment caused obesity and impaired glucose tolerance (i.e., T2DM conditions) in both wild‐type and *App^NL−F/NL−F^* mice, but only the latter animals exhibited an impaired cognitive function accompanied by marked increases in both Aβ deposition and microgliosis as well as insulin resistance in the hippocampus. Furthermore, HFD‐fed *App^NL−F/NL−F^* mice exhibited a significant decrease in volume of the granule cell layer in the dentate gyrus and an increased accumulation of 8‐oxoguanine, an oxidized guanine base, in the nuclei of granule cells. Gene expression profiling by microarrays revealed that the populations of the cell types in hippocampus were not significantly different between the two mouse lines, regardless of the diet. In addition, HFD treatment decreased the expression of the Aβ binding protein transthyretin (TTR) in *App^NL−F/NL−F^* mice, suggesting that the depletion of TTR underlies the increased Aβ deposition in the hippocampus of HFD‐fed *App^NL−F/NL−F^* mice.

## Graphical abstract

Insulin resistance and diabetes mellitus are major risk factors for Alzheimer's disease (AD). In this paper, we showed that type 2 diabetic conditions induced by chronic high‐fat diet treatment exacerbated pre‐existing AD pathology such as Aβ deposition, microgliosis, and oxidative DNA damage in a knock‐in mouse model of AD, *App* ^*NL‐F/NL‐F*^. This was accompanied by decreased expression of transthyretin, a protein that can bind to the Aβ peptide, suppress its aggregation, and promote its clearance.

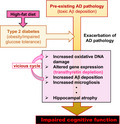

## 1 INTRODUCTION

Dementia is a syndrome that affects memory, thinking, behavior, and the ability to perform everyday activities (Ninomiya, 2019). Alzheimer's disease (AD) is a major type of neurodegenerative dementia, accounting for more than 70% of all cases (Gale et al., 2018). AD is characterized by two well‐known pathological hallmarks in the brain, the aggregation of β amyloid (Aβ) into plaques and the aggregation of hyperphosphorylated tau protein into neurofibrillary tangles (Selkoe, 2000). It has been over 100 years since the discovery of AD; however, its etiology is still not fully understood. Genetic factors such as mutations in the *APP*, *PSEN1*, and *PSEN2* genes only account for approximately 1% of AD cases, known as early‐onset or familial AD (FAD). In the majority of AD cases in which the onset is late or sporadic, aging is the main risk factor, and mounting epidemiological evidence has suggested that people with type‐2 diabetes mellitus (T2DM) or impaired glucose tolerance are at an increased risk for the development of AD (Arvanitakis et al., 2004; de la Monte, 2014; Ninomiya, 2019). Pathological studies have also shown a significant association between T2DM‐related factors and the neuropathology of AD, such as Aβ plaque formation (Matsuzaki et al., 2010; Peila et al., 2002). The etiology of cognitive dysfunction in AD patients with T2DM is probably multifactorial, but the mechanisms underpinning this association are not yet fully understood.

High‐fat diet (HFD) treatment is an established method to induce T2DM in experimental animals and has been applied to many AD mouse models. HFD treatment causes obesity and induces insulin resistance and impaired glucose metabolism in both the periphery and brain (Heydemann, 2016). Studies with AD mouse models have generally been performed using transgenic mice to induce Aβ accumulation and deposition by overexpressing a mutant amyloid precursor protein (*APP*) transgene, or with a combination of mutant *APP* and *PSEN1* or *MAPT* transgenes. Some studies with 3xTg‐AD mice carrying mutant *APP* and *MAPT* transgenes together with a *Psen1* knock‐in mutation have shown that HFD treatment increases brain Aβ levels with an exacerbated cognitive decline (Barron et al., 2013; Vandal et al., 2014). Other studies with 3xTg‐AD mice found an exacerbated cognitive decline without alteration of the Aβ levels (Knight et al., 2014; Sah et al., 2017). Some studies with *APP^Swe^/PS1^deltaE9^* mice have also reported that HFD treatment increases the brain levels of Aβ with an exacerbated cognitive decline (Ettcheto et al., 2016; Walker et al., 2017), while others with *APP^Swe^/PS1^deltaE9^* mice reported that HFD treatment increases the Aβ load but does not enhance the cognitive decline (Bracko et al., 2020; Yeh et al., 2015). Moreover, it has been reported that an HFD protects the blood–brain barrier in Tg2576 mice carrying the *APP^Swe^* transgene (Elhaik Goldman et al., 2018). Thus, there have been some controversies in the results obtained using transgenic AD mouse models. These contrasting results may be attributed to artificial effects of the transgenes introduced, such as their overexpression and ectopic expression.

A recent study using a novel knock‐in mouse model of AD, *App^NL/NL^* carrying the Swedish “NL” mutation, showed that chronic HFD treatment does not trigger AD‐associated pathological alterations, such as hippocampal amyloidosis, Tau phosphorylation, and cognitive impairment, although mild impairments in both hippocampal long‐term potentiation and social memory were observed (Salas et al., 2018). Despite carrying a FAD mutation, this mouse line does not spontaneously develop Aβ deposition. Given the findings from transgenic mouse models of AD mice with HFD treatment, we hypothesized that prior Aβ deposition may be a prerequisite for HFD treatment to exacerbate the development or progression of AD. It is reasonable to assume that T2DM has an impact on amyloidosis since Aβ deposition starts at 10–20 years before the clinical onset of AD (Bateman et al., 2012), and T2DM increases the risk of AD development during this early stage without clinical symptoms of AD (Ninomiya, 2014).

To test this hypothesis, we applied HFD treatment to an *App^NL−F/NL−F^* knock‐in mouse model of AD, which carries a humanized β‐amyloid (Aβ) sequence with two pathogenic mutations: Swedish “NL” and Iberian “F” at the authentic mouse *App* locus, thereby increasing the production of the pathogenic Aβ without the overproduction of APP. *App^NL−F/NL−F^* mice accumulate Aβ plaques from 6 months of age and show very mild cognitive impairment at 18 months of age. The present study provides the first experimental evidence demonstrating that T2DM exacerbates pre‐existing AD pathology, and that AD pathology does not induce T2DM in regular diet (RD)‐fed *App^NL−F/NL−F^* mice.

## 2 RESULTS

### 2.1 An HFD altered the metabolic parameters and brain insulin signaling in both wild‐type and *App^NL−F/NL−F^* mice

**FIGURE 1:**
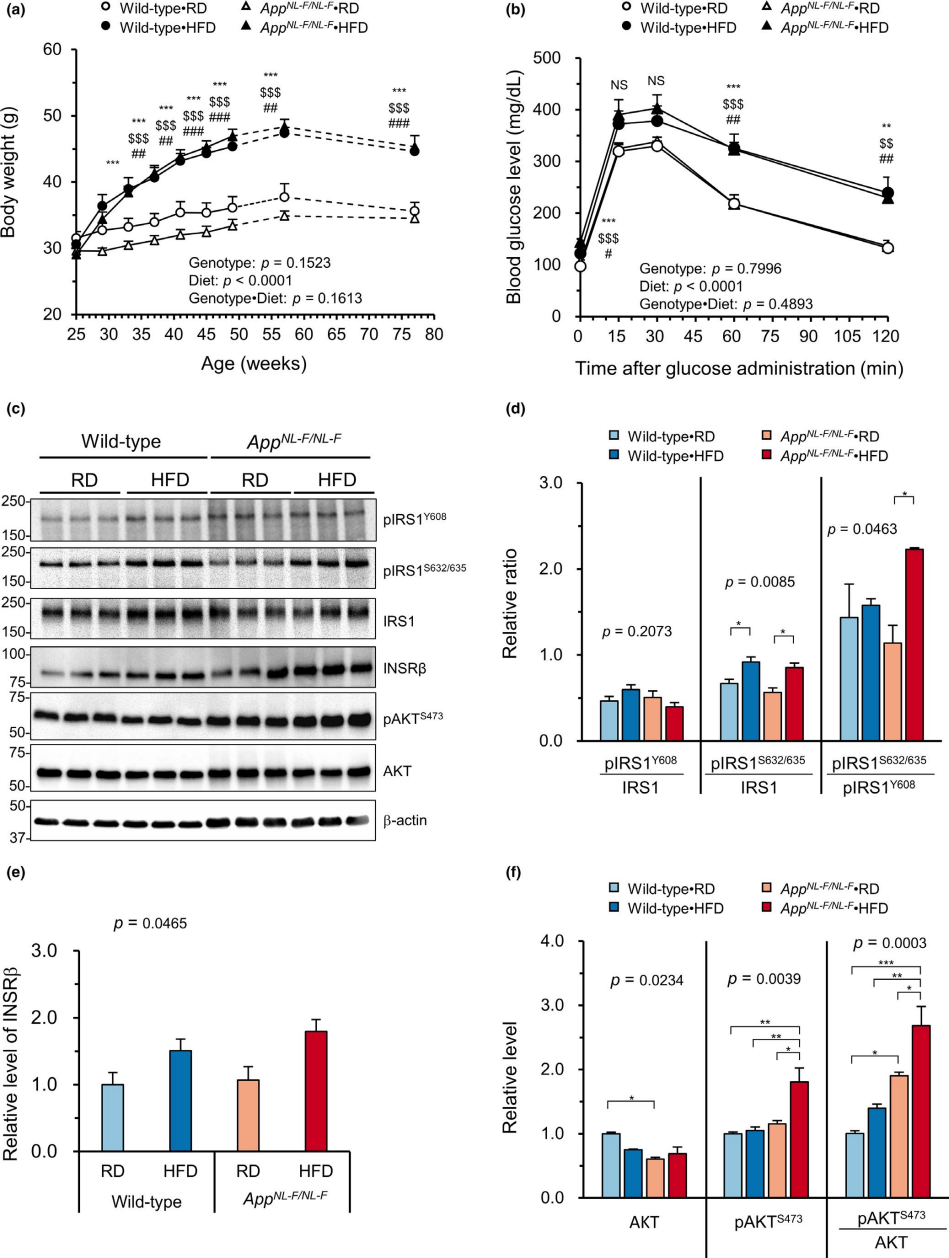
A high‐fat diet led to increased body weight, impaired glucose metabolism, and an impaired insulin signaling pathway in both wild‐type and *App^NL−F/NL−F^* mice. (a) Six‐month‐old wild‐type and *App^NL−F/NL−F^* mice were fed either a regular (RD) or high‐fat diet (HFD), and their weights were plotted every 4 weeks for a period of 24 weeks (solid line) and then at 58 and 78 weeks of age (dotted line). (b) Intraperitoneal glucose tolerance test at 18 months of age. After 6 h of fasting, mice were injected with glucose (2 g/kg of body weight). Blood glucose levels were then monitored over time. (c) Representative Western blots showing the hippocampal levels of insulin signaling proteins (pIRS1^Y608^, pIRS1^S632/635^, IRS1, INSRβ, pAKT^S473^, and AKT) in *App^NL−F/NL−F^* and wild‐type mice fed an RD or HFD. (d) Relative ratios of pIRS1^Y608^/IRS1, pIRS1^S632/635^/IRS1, and pIRS1^S632/635^/pIRS1^Y608^ in the blots using β‐actin as a loading control. (e) Quantification of protein levels of INSRβ in the blot using β‐actin as a loading control. (f) Quantification of the protein levels of pAKT^S473^ and AKT and the pAKT^S473^/AKT ratio in the blots using β‐actin as a loading control. The bar graph shows the protein/β‐actin ratio relative to RD‐fed wild‐type mice. The data were expressed as the mean ± SEM, *n* = 17 (solid line) or 10 (dotted line) for RD‐fed wild‐type mice (Wild‐type•RD), *n* = 12 (solid line) or 5 (dotted line) for HFD‐fed wild‐type mice (Wild‐type•HFD), *n* = 29 (solid line) or 13 (dotted line) for RD‐fed *App^NL−F/NL−F^* mice (*App^NL−F/NL−F^* •RD), and *n* = 22 (solid line) or 12 (dotted line) for HFD‐fed *App^NL−F/NL−F^* mice (*App^NL−F/NL−F^* •HFD) in (a); *n* = 4–5 for all groups in (b). *n* = 3 for all groups in (d–f). The results were statistically analyzed by a MANOVA (*p* values for effects are shown) with data from 25 to 49 weeks of age in (a) or 0 to 120 min in (b), and a two‐way ANOVA followed by post hoc Tukey's Honest Significant Difference (HSD) test was applied to the data for each week, ^**^ *p* < 0.01 and ^***^ *p* < 0.001 for *App^NL−F/NL−F^* •HFD vs. *App^NL−F/NL−F^* •RD; ^$$^ *p* < 0.01 and ^$$$^ *p* < 0.001 for *App^NL−F/NL−F^* •HFD vs. Wild‐type•RD; and ^#^ *p* < 0.05, ^##^ *p* < 0.01, ^###^ *p* < 0.001 for Wild‐type•HFD vs. Wild‐type•RD. NS, not significant. Weeks 58 and 78 in (a) were excluded from the two‐way repeated measures ANOVA and instead subjected to a one‐way ANOVA with post hoc Tukey's HSD test. The results in (d–f) were statistically analyzed by a two‐way ANOVA (*p* values for each analysis shown) followed by post hoc Tukey's HSD test, ^*^ *p* < 0.05, ^**^ *p* < 0.01 and ^***^ *p* < 0.001

To examine whether or not *App^NL−F/NL−F^* mice respond to HFD treatment in a similar way to wild‐type mice, wild‐type and *App^NL−F/NL−F^* male mice were fed either an RD or an HFD for 12 months starting at 6 months (25 weeks) of age. As shown in Figure 1a, HFD treatment significantly increased the body weights in both genotypes compared to the RD‐fed groups (Tukey's HSD test, *p* < 0.0001). There were no phenotypical changes between the HFD‐fed wild‐type and *App^NL−F/NL−F^* mice, and their increased body weights were maintained throughout the experiment. Both genotypes of mice fed an HFD exhibited a significantly lower intake of food and water in comparison with those fed an RD (Figure S1a,b); however, the calorie intake calculated from the amounts of diet consumed was significantly increased in both genotypes of mice fed an HFD. To evaluate the effect of an HFD on their glucose metabolism, we measured the fasting blood glucose levels every other week up to 49 weeks of age. As expected, HFD treatment significantly increased fasting blood glucose levels from the second week of treatment in both genotypes of mice (Figure S1c). To examine whether HFD treatment induces a diabetic condition, mice were subjected to an intraperitoneal glucose tolerance test (IPGTT) at 18 months of age. As shown in Figure 1b, HFD treatment caused significantly impaired glucose tolerance, regardless of the genotype, indicating that an HFD increases body weight and disrupts glucose metabolism in both wild‐type and *App^NL−F/NL−F^* mice.

Subsequently, we assessed the hippocampal insulin signaling by Western blotting (Figure 1c). Consistent with the peripheral glucose metabolism impairment, we found an increase in basal levels of IRS1 phosphorylation at Ser^632/635^, which negatively regulates the IRS1 function by reducing its association with PI3‐kinase, in both wild‐type and *App^NL−F/NL−F^* mice fed an HFD, while the basal level of IRS1 phosphorylation at Tyr^608^, which generates a docking site for the PI3‐kinase, was slightly decreased only in the HFD‐fed *App^NL−F/NL−F^* mice, thereby significantly increasing the pIRS1^S632/635^/pIRS1^Y608^ ratio (Figure 1d). The total protein levels of insulin receptor β (INSRβ) and AKT were not significantly altered by the diet in either genotype, but the basal level of AKT phosphorylation at Ser^473^ was significantly increased only in the HFD‐fed *App^NL^* ^−^ *^F^* ^/^ *^NL^* ^−^ *^F^* mice (Figure 1e,f). The hyperphosphorylation of AKT is known to increase phosphorylation of serine residues of IRS1 through activation of mTOR pathway (Copps & White, 2012). These results suggest that HFD induces a more intense hippocampal insulin resistance in *App^NL−F/NL−F^* mice than in wild‐type mice.

### 2.2 An HFD impaired the cognitive function only in *App^NL−F/NL−F^* mice

**FIGURE 2:**
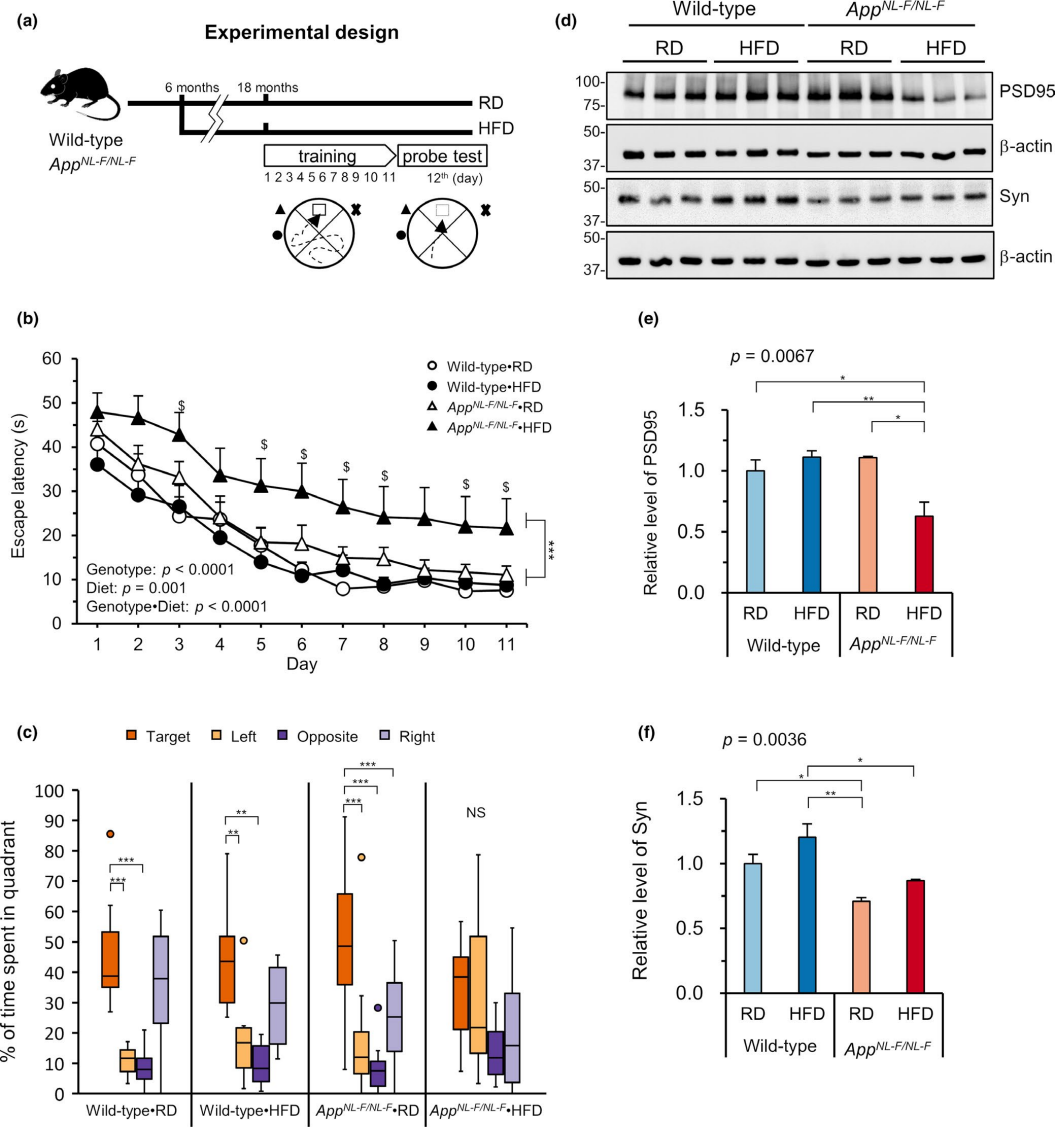
High‐fat diet treatment impaired spatial learning and levels of post‐synaptic markers in *App^NL−F/NL−F^* mice. (a) Diagram of the experimental design for the behavioral test. Six‐month‐old wild‐type and *App^NL−F/NL−F^* mice were fed either a regular diet (RD) or high‐fat diet (HFD). When they reached 18 months of age, spatial learning and memory recall were tested by a Morris Water Maze test (MWM). (b) The MWM test showed a significantly impaired spatial learning ability in the *App^NL−F/NL−F^* mice fed an HFD (*App^NL−F/NL−F^* •HFD) compared with all three other groups. (c) Percentage of the time spent in each quadrant during the probe test, 24 h after the last training. *App^NL−F/NL−F^* •HFD mice, but not other three groups of mice, showed no preference for the target quadrant among the four quadrants. (d) Western blots showing the hippocampal levels of PSD95 and synaptophysin (Syn) in *App^NL−F/NL−F^* and wild‐type mice fed an RD or HFD. (e–f) Quantification of protein levels in blots using β‐actin as a loading control. The bar graph shows the protein/β‐actin ratio relative to RD‐fed wild‐type mice. Data are expressed as the mean ± SEM, *n* = 11–14 for all groups for (b–c) and *n* = 3 for (e–f). Statistical analyses for (b) were performed by a two‐way repeated measures ANOVA (*p* values for effects are shown) where ^***^ *p* < 0.001 for *App^NL−F/NL−F^* •HFD vs. all three other groups, followed by post hoc Tukey's HSD test, where ^$^ *p* < 0.05 for *App^NL−F/NL−F^* •HFD vs. Wild‐type•RD. A nonparametric comparison with the % time spent in the target quadrant performed using the Steel method (c), where ^***^ *p* < 0.001. For (e, f), a two‐way ANOVA (*p* values for each analysis shown) was performed followed by post hoc Tukey's HSD test, ^*^ *p* < 0.05, ^**^ *p* < 0.01

We next examined whether HFD treatment impairs the cognitive function by performing a Morris water maze test at 18 months of age. Mice were trained to find a hidden platform underwater for 11 consecutive days and then subjected to a probe test without a platform (Figure 2a). During training, *App^NL−F/NL−F^* mice fed an HFD showed a significantly increased (*p* < 0.0001) escape latency to the platform in comparison with all other groups, with significant effects of genotype, diet, and interaction between genotype and diet (Figure 2b). During the probe test at 24 h after the last training, *App^NL−F/NL−F^* •HFD mice, but not the other three groups of mice, exhibited no preference for the target quadrant among all quadrants (Figure 2c), indicating that HFD administration significantly impairs memory retrieval only in *App^NL−F/NL−F^* mice. Furthermore, the frequency of virtual platform crossing was decreased, and the time to the target quadrant was increased in *App^NL−F/NL−F^* mice fed an HFD compared to all other groups; however, the differences did not reach statistical significance (Figure S2a,b). The swimming speed did not differ markedly among the groups (Figure S2c). Next, we examined the levels of pre‐ and post‐synaptic proteins in the hippocampus by Western blotting (Figure 2d). As shown in Figure 2e, the levels of post‐synaptic PSD95 were significantly decreased in the HFD‐fed *App^NL−F/NL−F^* mice compared to the other groups, and the levels of pre‐synaptic synaptophysin were significantly decreased in *App^NL−F/NL−F^* mice regardless of the diet compared to wild‐type mice (Figure 2f). These results indicate that an HFD disturbs synaptic integrity only in *App^NL−F/NL−F^* mice.

### 2.3 An HFD increased Aβ deposition in *App^NL−F/NL−F^* mice

**FIGURE 3:**
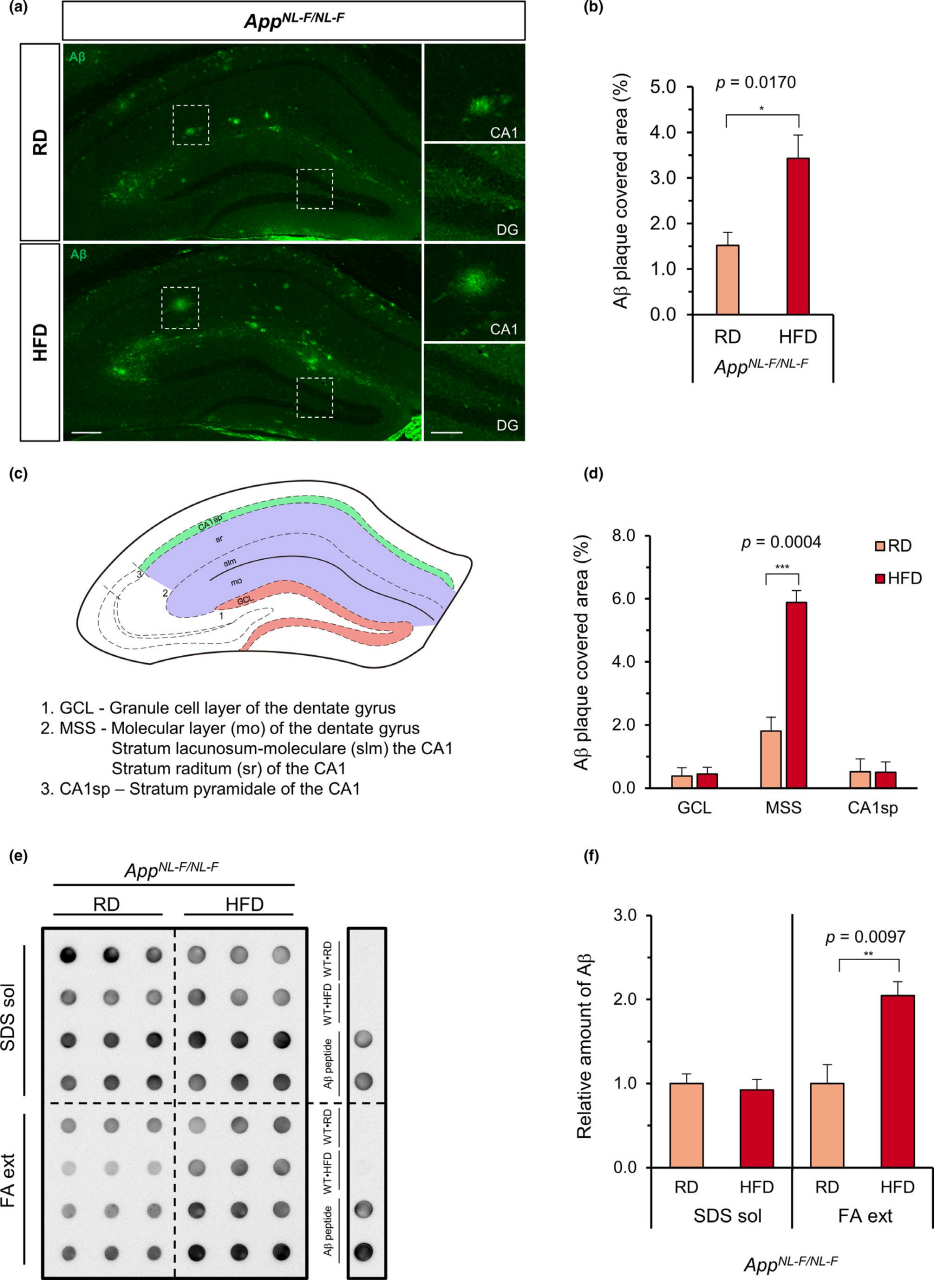
High‐fat diet treatment increased the amyloid‐β deposition in the hippocampus of *App^NL−F/NL−F^* mice. (a) Representative immunofluorescence images of Aβ in the hippocampus of *App^NL−F/NL−F^* mice fed a regular diet (RD) or high‐fat diet (HFD). Aβ plaques were stained using anti‐Aβ 82E1(green) and subsequentially quantified. No plaques were detected in the wild‐type mice (Figure S3). Scale bar = 200 µm (for full image) and 100 µm (for augmented areas), DG =dentate gyrus. (b) Quantification of the Aβ plaque‐covered area in the whole hippocampus. (c) Diagram depicting the 3 studied zones in the hippocampus; granule cell layer of DG (GCL), the molecular layer of DG plus the stratum lacunosum moleculare—stratum radiatum of CA1 (MSS), and the stratum pyramidale of CA1 (CA1sp) are shown. (d) Quantification of the Aβ plaque‐covered area in 3 different zones of the hippocampus. The Aβ plaque‐covered area is shown as bar graphs. (e) Aβ dot blotting of the SDS‐soluble (SDS sol.) and formic acid‐extractable (FA ext.) protein fractions of hippocampal tissue of *App^NL−F/NL−F^* mice fed an RD or HFD. Three dots in each row were from the same sample, and the blot was reacted with anti‐Aβ 82E1. Wild‐type•RD (WT•RD), Wild‐type•HFD (WT•HFD), and Aβ peptide were used as controls. No Aβ was detected in the wild‐type samples. (f) Quantification of the Aβ in the dot blot. The values are relative to the integrated density of the dot blot observed in RD‐fed *App^NL−F/NL−F^* mice. The mean intensities were normalized to Ponceau S staining (SDS sol) or total protein (FA ext). Data expressed as the mean ± SEM, *n* = 4 for all experiments. Four brain slices per mouse were examined for (b) and (d). The results were statistically analyzed by an unpaired *t* test, ^*^ *p* < 0.05, ^**^ *p* < 0.01, and ^***^ *p* < 0.001

We next assessed Aβ deposition by immunofluorescence microscopy with an anti‐Aβ (82E1) antibody (Figure 3a). Quantification of the Aβ plaque‐covered area revealed that *App^NL−F/NL−F^* mice fed an HFD showed a significantly increased (more than twofold) Aβ plaque‐covered area in the hippocampus in comparison with *App^NL−F/NL−F^* mice fed an RD (Figure 3b). No plaques were found in the wild‐type mice (Figure S3a). It is noteworthy that in *App^NL−F/NL−F^* mice fed an HFD, most of the plaques were located in the central zone of the hippocampus, namely in the area between the pyramidal cell layer of CA1 and the dentate gyrus (DG) (Figure 3a).

We then compared the degree of the Aβ plaque coverage in 3 defined zones in the hippocampus, as shown in the schematic illustration in Figure 3c: the granule cell layer of the DG (GCL), the upper part of the molecular layer of the DG plus the stratum lacunosum moleculare and stratum radiatum of the CA1 (MSS), and the stratum pyramidale of the CA1 (CA1sp). We found that in *App^NL−F/NL−F^* •RD mice, the MSS exhibited the highest degree of Aβ plaque coverage (approximately 2%) among the three zones, and HFD treatment significantly and selectively increased it to approximately 6% (Figure 3d). We then quantified the SDS‐soluble (SDS sol.) and SDS‐insoluble/formic acid‐extractable (FA ext.) Aβ present in the hippocampal protein fractions with a dot blot analysis using anti‐Aβ (82E1) antibody (Figure 3e,f). We found that *App^NL−F/NL−F^* mice fed an HFD had a significantly (*p* = 0.009) higher amount of FA‐extractable Aβ than *App^NL−F/NL−F^* mice fed an RD, while the levels of SDS‐soluble Aβ were not affected by diet. We also examined the phosphorylated Tau accumulation by immunohistochemistry using an anti‐Tau (AT8) antibody. No positive signal was detected in *App^NL−F/NL−F^* mice fed an RD or those fed an HFD (Figure S3b).

To clarify whether or not HFD treatment affects either APP processing or Aβ formation, we examined the levels of APP and APP‐derived fragments by Western blotting (Figure S4a). *App^NL−F/NL−F^* mice fed an HFD showed a significant decrease in full‐length APP (FL‐APP) detected by the anti‐APP A4 (22C11) that recognizes N‐terminal residues (a.a. 66–81) of APP. Two other antibodies (6E10, APP‐CT) also showed that an HFD caused a similar but not significant decrease in FL‐APP in *App^NL−F/NL−F^* mice. In contrast, an HFD slightly increased FL‐APP in wild‐type mice (Figure S4b–d). Humanized Aβ detected by 6E10 was only found in *App^NL−F/NL−F^* mice with no diet effect (Figure S4e). Accordingly, significantly higher levels of β secretase‐cleaved CTFβ fragment were detected in *App^NL−F/NL−F^* mice than in wild‐type mice, and they were slightly increased by an HFD (Figure S4f). In contrast, slightly lower levels of α secretase‐cleaved CTFα were detected in *App^NL−F/NL−F^* mice than in wild‐type mice, with a slight decrease due to an HFD (Figure S4g). These results suggest that APP processing by β secretase to generate Aβ may be slightly but not significantly enhanced by an HFD in *App^NL−F/NL−F^* mice.

Next, we examined the hippocampal gene expression profiles of all four groups of mice using a microarray analysis of hippocampal RNA and confirmed no significant difference in the expression of genes involved in APP processing and Aβ clearance (Table S1). Taken together, these results showed that an HFD did not significantly alter the APP processing or Aβ clearance in *App^NL−F/NL−F^* mice.

### 2.4 An HFD increased the hippocampal expression of genes involved in glial activation in *App^NL−F/NL−F^* mice

Next, we compared the expression of specific marker genes for five major types of brain cells: astrocytes, oligodendrocytes, microglia, neural stem cells/progenitor cells (NSCs/NPCs), and neurons to assess the cell population changes caused by diet and genotype (Table 1). We observed an increased expression of markers related to microglial (*C1qa*, *C1qb*, and *Cd68*) and astrocytic (*Gfap*) activation in *App^NL−F/NL−F^* mice fed an RD or HFD and also in HFD‐fed wild‐type mice in comparison with RD‐fed wild‐type mice. *App^NL−F/NL−F^* mice fed an HFD exhibited the highest expression of these genes, suggesting that an HFD exacerbates glial activation in the *App^NL−F/NL−F^* hippocampus, but has a limited effect in the wild‐type hippocampus. No differences were observed among the 4 groups in the expression of markers related to the three other cell types (neuron, NSC/NPC, and oligodendrocyte) (Table 1).

**TABLE 1:** The altered expression of marker genes for various brain cell types in the hippocampi of wild‐type and *App^NL−F/NL−F^* mice fed an RD or HFD

Cell type	Marker gene	Relative expression (% RD‐fed wild‐type mice)		
		HFD‐fed wild‐type	RD‐fed *App^NL−F/NL−F^*	HFD‐fed *App^NL−F/NL−F^*
Astrocyte	*Aqp4*	96.42	120.13	107.82
	*Gfap*	102.86	115.10	128.01
	*Glul*	99.98	107.68	108.20
	*S100b*	94.57	110.69	107.27
	*Slc1a1*	99.27	104.44	104.10
	*Slc1a2*	98.19	109.86	99.00
	Mean	98.55	111.32	109.07
	SD	2.88	5.57	9.90
Oligodendrocyte	*Mag*	112.78	103.41	124.33
	*Mbp*	107.55	108.34	111.51
	*Mog*	110.97	98.34	115.80
	*Sox10*	111.30	102.11	109.95
	Mean	110.65	103.05	115.40
	SD	2.21	4.13	6.45
Microglia	*Aif1*	100.60	98.21	94.12
	*C1qa*	121.59	130.97	139.62
	*C1qb*	116.39	120.24	132.32
	*Cd68*	128.68	145.24	165.39
	*Itgam*	98.89	96.71	104.91
	*Lgals3*	102.29	98.30	108.48
	*Ptprc*	117.32	108.44	145.07
	Mean	112.25	114.02	127.13
	SD	11.64	18.83	25.49
NSC/NPC	*Ascl1*	99.32	112.66	110.23
	*Bmp4*	111.59	127.28	160.18
	*Dcx*	105.75	119.54	96.37
	*Egf*	94.50	101.41	104.82
	*Igf2*	150.47	148.18	156.45
	*Notch2*	123.84	113.32	99.78
	*Nr2e1*	101.36	95.07	94.97
	*Shh*	110.79	104.09	109.80
	*Sox2*	98.13	106.47	94.87
	Mean	110.64	114.22	114.16
	SD	17.38	16.00	25.72
Neuron	*Chga*	95.33	101.57	103.23
	*Eno2*	92.32	92.81	93.68
	*Nefh*	88.86	103.59	91.82
	*Nefl*	92.69	100.71	100.92
	*Nefm*	88.67	110.27	99.87
	*Rbfox3*	94.75	90.48	93.11
	*Snap25*	94.34	102.92	100.44
	*Syp*	99.25	96.42	100.41
	*Syt1*	96.74	99.58	97.54
	*Tubb2a*	92.63	86.51	94.41
	Mean	93.56	98.49	97.54
	SD	2.95	8.61	2.69

Note

Significantly altered genes vs. RD‐fed wild‐type mice (eBayes ANOVA: *p* < 0.05) are underlined.

Abbreviations: HFD, high‐fat diet; NPC, neural progenitor cell; NSC, neural stem cell; RD, regular diet.

**FIGURE 4:**
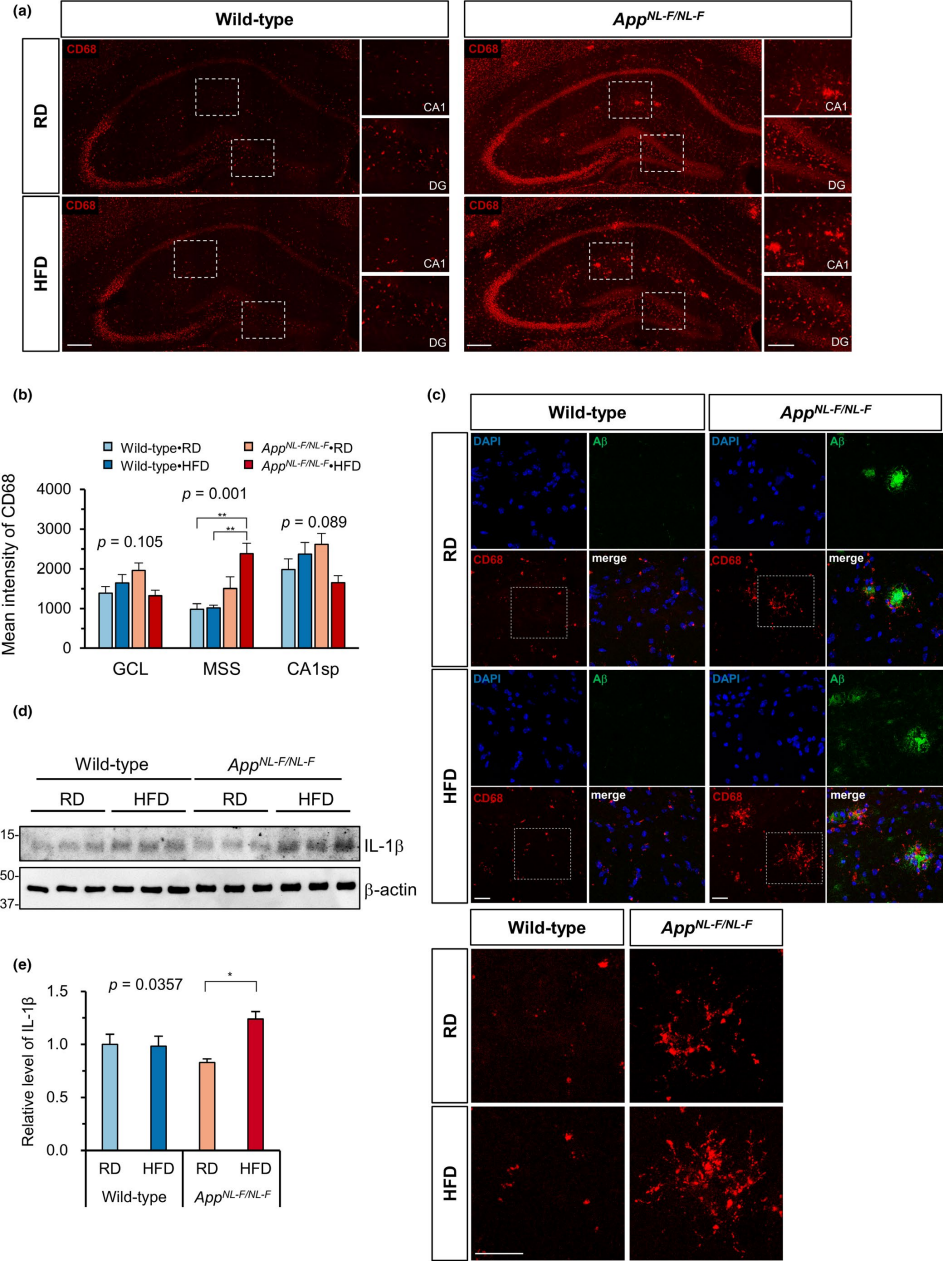
A high‐fat diet promoted microglial activation in the hippocampus of *App^NL−F/NL−F^* mice. (a) Representative immunofluorescence images of CD68‐positive microglial cells in the hippocampus of *App^NL−F/NL−F^* and wild‐type mice fed an RD or HFD. Scale bar = 200 µm (for full image) and 100 µm (for magnified images), DG, dentate gyrus. (b) Quantification of CD68 intensity in three different zones of the hippocampus shows the increased expression of CD68 in *App^NL−F/NL−F^* mice fed an HFD. (c) Z‐stack projections of multi‐immunofluorescence images for Aβ and CD68 shows that CD68‐positive microglia were clustered surrounding Aβ plaques (top). Magnified view of the dotted boxes for CD68 signal (bottom). Scale bar = 50 µm. (d) Western blots showing the hippocampal levels of IL‐1β in *App^NL−F/NL−F^* and wild‐type mice fed an RD or HFD. (e) Quantification of IL‐1β levels in blots using β‐actin as a loading control. The bar graph shows the IL‐1β/β‐actin ratio relative to RD‐fed wild‐type mice. Data are expressed as the mean ± SEM, *n* = 4 and four brain slices per mouse were examined for (b) and *n* = 3 for (e). The results were statistically analyzed by a two‐way ANOVA (*p* values for each analysis shown) followed by post hoc Tukey's HSD test, where ^*^ *p* < 0.05 and ^**^ *p* < 0.01

To confirm the activated state of glial cells in hippocampus, we performed immunofluorescence microscopy of coronal brain sections using anti‐CD68 antibody (Figure 4a). A much stronger CD68 immunoreactivity (IR) was detected in the hippocampus of RD‐fed *App^NL−F/NL−F^* mice than in that of RD‐fed wild‐type mice, and it was significantly increased by an HFD but only in the MSS zone of the *App^NL−F/NL−F^* mice (Figure 4b). Furthermore, multi‐immunofluorescence microscopy for Aβ and CD68 revealed that CD68‐positive cells were mostly located surrounding Aβ plaques in *App^NL−F/NL−F^* mice (Figure 4c). Western blotting of hippocampal extracts confirmed that an HFD significantly increased the levels of IL‐1β in only *App^NL−F/NL−F^* mice compared to the RD‐fed group (Figure 4d,e). Relatively strong GFAP IR was detected in the MSS zone of both the wild‐type and *App^NL−F/NL−F^* hippocampus with a tendency to be increased compared with the former. However, GFAP IR in the three zones of the hippocampus were not affected by diet in either genotype (Figure S5a,b). In *App^NL−F/NL−F^* mice, GFAP‐positive cells were mostly detected in areas surrounding Aβ plaques (Figure S5c). These results are consistent with the microarray data and indicate that an HFD is associated with an increase in Aβ deposition accompanied by microglial activation in the MSS zone of the hippocampus in *App^NL−F/NL−F^* mice.

### 2.5 An HFD increased nuclear accumulation of 8‐oxoguannine and altered gene expression in the hippocampus of *App^NL−F/NL−F^* mice

**FIGURE 5:**
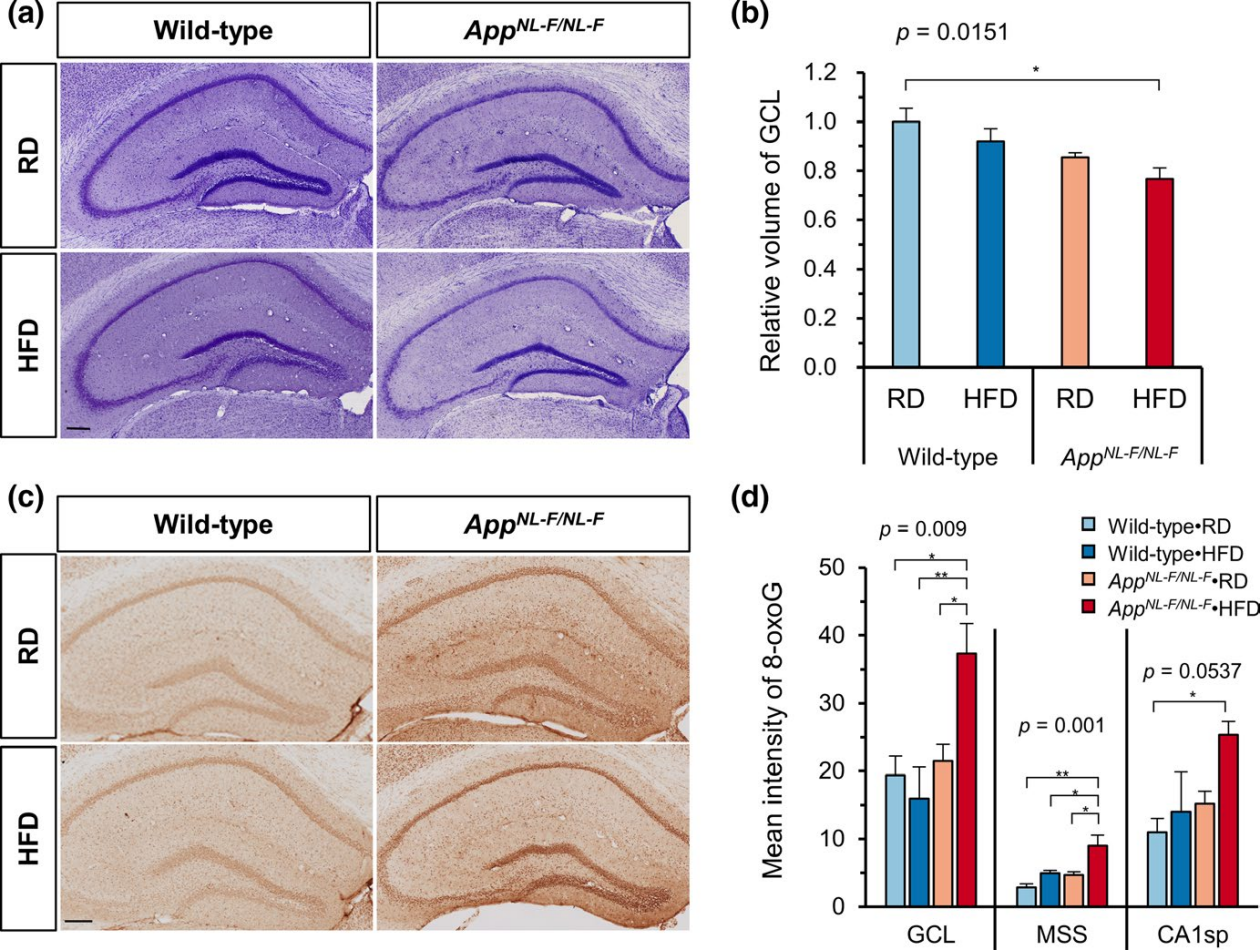
A high‐fat diet increased nuclear 8‐oxoguanine accumulation in granule cells and reduced the volume of the granule cell layer in the hippocampus of *App^NL−F/NL−F^* mice. (a) Representative images of the Nissl‐stained hippocampus of *App^NL−F/NL−F^* and wild‐type mice fed an RD or HFD. Scale bar = 200 µm. (b) GCL volume estimation using the Cavalieri method with 5 serial sections per brain showed that the volume of GCL was decreased in *App^NL−F/NL−F^* mice fed HFD. (c) Representative images of the immunohistochemical detection of 8‐oxoG in nuclear DNA in the hippocampus of *App^NL−F/NL−F^* and wild‐type mice fed an RD or HFD. Scale bar = 200 µm. (d) The quantitative measurement of the 8‐oxoG intensity in nuclear DNA in 3 zones of hippocampus: GCL, MSS, and CA1sp. Data expressed as the mean ± SEM, *n* = 4, and four brain slices were examined per mouse. The results were statistically analyzed by a two‐way ANOVA (*p* values for each analysis shown) followed by post hoc Tukey's HSD test, ^*^ *p* < 0.05, and ^**^ *p* < 0.01

Amyloid deposition and glial activation are known to induce oxidative stress, which causes the oxidation of various molecules, including DNA, resulting in cellular dysfunction (Nakabeppu, 2019). This raises a question as to whether an HFD increases oxidative stress and cellular damage in the *App^NL−F/NL−F^* hippocampus. To examine the extent of cellular damage in the hippocampus, we performed cresyl violet staining of brain sections from wild‐type and *App^NL−F/NL−F^* mice fed an RD or HFD (Figure 5a). We found that *App^NL−F/NL−F^* mice tended to show a reduced GCL volume in comparison with wild‐type mice (Student's *t* test, *p* = 0.0484) and that HFD treatment also decreased the volume in both genotypes of mice to some extent. As a result, HFD‐fed *App^NL−F/NL−F^* mice exhibited a significantly reduced GCL volume in comparison with RD‐fed wild‐type mice (2‐way ANOVA, Tukey's HSD post hoc test, *p* = 0.0151) (Figure 5b). These findings were consistent with an exacerbated AD‐like pathology in the HFD‐fed *App^NL−F/NL−F^* mice. Of note, Fluoro‐Jade C staining showed no sign of degenerating neurons in the hippocampus of any group (Figure S6a,b).

To evaluate the extent of oxidative stress in the hippocampus, we examined the accumulation of the oxidized guanine base 8‐oxoguanine (8‐oxoG) in brain sections from wild‐type and *App^NL−F/NL−F^* mice fed an RD or HFD using immunohistochemistry with anti‐8‐oxo‐2´‐deoxyguanosine (8‐oxo‐dG) antibody (Figure 5c). We found a slightly higher 8‐oxoG IR in the hippocampus of RD‐fed *App^NL−F/NL−F^* mice than in RD‐fed wild‐type mice, which was significantly increased by HFD treatment. Quantification of 8‐oxoG IR revealed that HFD‐fed *App^NL−F/NL−F^* mice exhibited the highest 8‐oxoG IR in the three zones of the hippocampus among all groups (Figure 5d). The 8‐oxoG IR in the GCL and MSS of the HFD‐fed *App^NL−F/NL−F^* hippocampus was significantly higher than the IR found in all other groups, but the value in CA1sp showed a significant increase only compared to RD‐fed wild‐type mice. In addition, the levels of 4‐hydroxynonenal (4‐HNE) modified proteins in hippocampus were examined by Western blotting to evaluate the extent of lipid peroxidation‐related damage. *App^NL−F/NL−F^* mice showed significantly increased levels of 4‐HNE‐modified proteins compared to wild‐type mice, but the levels were not affected by HFD treatment in either genotype (Figure S6c,d). Furthermore, the hippocampal expression of antioxidant genes was also not affected by either diet or genotype (Table S2). These results suggest that HFD treatment resulted in increased Aβ deposition and neuroinflammation in the MSS and oxidative DNA damage in both the MSS and GCL of the hippocampus, thereby reducing the GCL volume.

**FIGURE 6:**
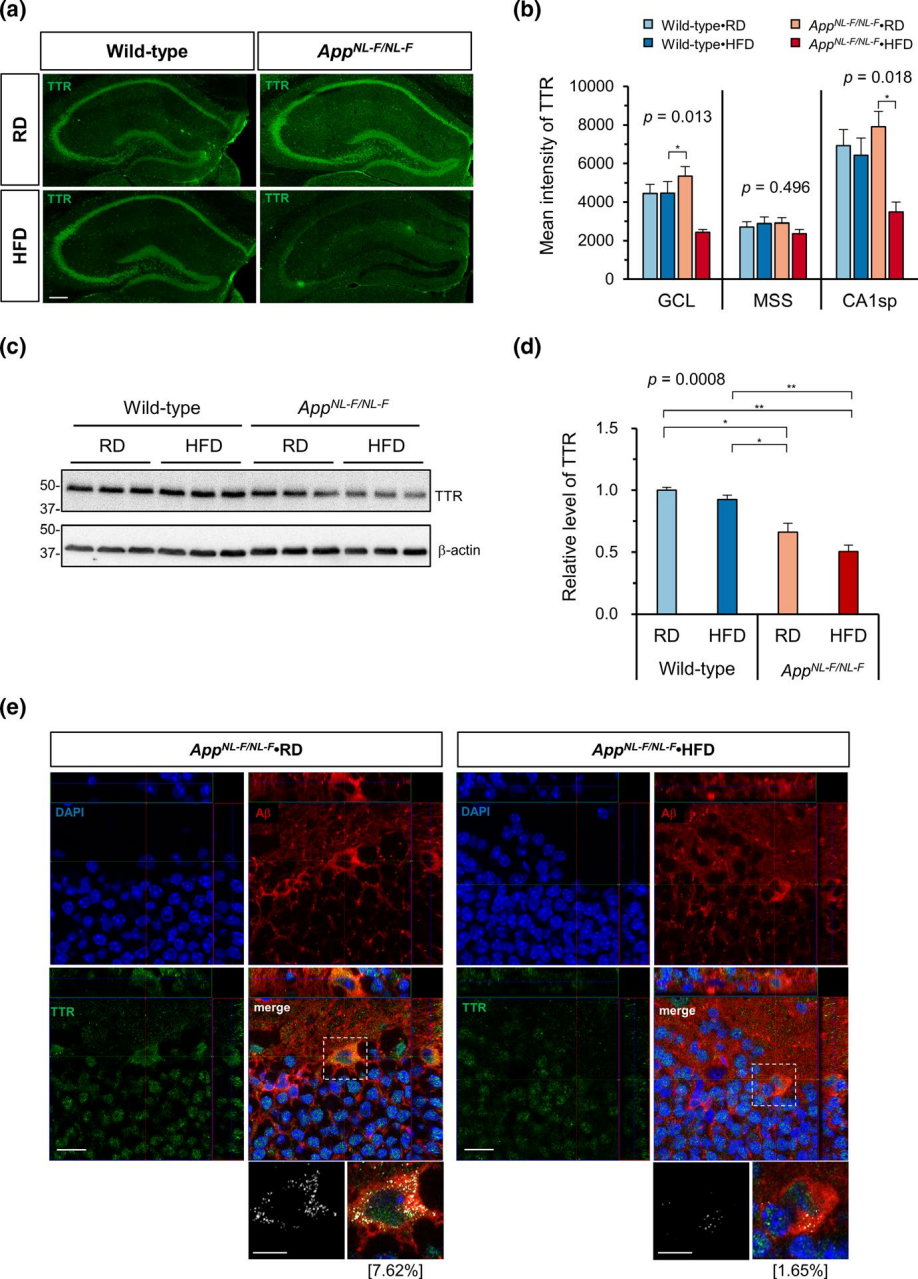
A high‐fat diet decreased the transthyretin expression in the hippocampus of *App^NL−F/NL−F^* mice. (a) Representative immunofluorescence images of TTR in the hippocampus of *App^NL−F/NL−F^* and wild‐type mice fed an RD or HFD. Scale bar = 200 µm. (b) The quantitative measurement of the TTR intensity in three zones of the hippocampus (GCL, MSS, and CA1sp) revealed the decreased expression of TTR in *App^NL−F/NL−F^* mice fed an HFD. (c) Representative Western blots showing the hippocampal levels of TTR in *App^NL−F/NL−F^* and wild‐type mice fed an RD or HFD. (d) Quantification of TTR protein levels in blots using β‐actin as a loading control. The bar graph shows the TTR/β‐actin ratio relative to RD‐fed wild‐type mice. (e) Orthogonal view of multi‐immunofluorescence microscopy for Aβ and TTR shows the intracellular TTR signal co‐localized with Aβ in *App^NL−F/NL−F^* mice fed an RD. In magnified bitmap images from the dotted boxes (bottom), the co‐localized red and green pixels are shown in white. The percentage of the co‐localized pixels among total TTR pixels is shown in brackets. Scale bar = 20 µm for full images and 10 µm for magnified images. Data are expressed as the mean ± SEM, *n* = 3 for all experiments, and 3 brain slices per mouse were examined for (b). The results were statistically analyzed by a two‐way ANOVA (*p* values are shown for each analysis) followed by post hoc Tukey's HSD test, ^*^ *p* < 0.05, and ^**^ *p* < 0.01

To delineate the molecular effects of HFD treatment in the *App^NL−F/NL−F^* hippocampus, we compared the gene expression profiles of RD‐fed *App^NL−F/NL−F^* and HFD‐fed *App^NL−F/NL−F^* mice. As shown in the Table S3, HFD treatment significantly altered the expression of several genes (±1.5‐fold change, minimum raw intensity >100) in the hippocampus. We found that HFD treatment of *App^NL−F/NL−F^* mice tended to decrease the expression of *Ttr*, a gene encoding the amyloid binding protein transthyretin (TTR), to 15% or 19% of the levels seen in wild‐type or RD‐fed *App^NL−F/NL−F^* mice, respectively. However, the change did not reach statistical significance. As a result, we examined the levels of TTR in hippocampus by immunofluorescence microscopy using an anti‐TTR antibody. As shown in Figure 6a, HFD‐fed *App^NL−F/NL−F^* mice exhibited the lowest TTR IR in the hippocampus among all groups. Quantification of the TTR IR revealed that HFD treatment of *App^NL−F/NL−F^* mice significantly decreased the levels of TTR in both the GCL and the CA1sp zones but not in the MSS zone of the hippocampus (Figure 6b). Quantification of the TTR IR in the cortex also revealed that HFD treatment decreased the cortical levels of TTR in *App^NL−F/NL−F^* mice (Figure S7a,b). In addition, we examined the total hippocampal levels of TTR by Western blotting (Figure 6c). We confirmed that HFD‐fed *App^NL−F/NL−F^* mice had the lowest level of hippocampal TTR among all groups, and that this level was significantly lower than in RD‐fed or HFD‐fed wild‐type mice (Figure 6d). Finally, multi‐immunofluorescence microscopy for Aβ and TTR demonstrated the intracellular co‐localization of Aβ and TTR in the GCL of RD‐fed *App^NL−F/NL−F^* mice and its apparent reduction by HFD treatment (Figure 6e).

## 3 DISCUSSION

The major conclusion of the present study is that chronic HFD treatment caused obesity and impaired glucose tolerance (i.e., induced T2DM conditions) in both wild‐type and *App^NL−F/NL−F^* mice, with only the latter exhibiting an impaired cognitive function accompanied by marked increases in both Aβ deposition and microgliosis as well as insulin resistance in the hippocampus. Moreover, HFD‐fed *App^NL−F/NL−F^* mice exhibited a significant decrease in GCL volume in the DG and an increased accumulation of 8‐oxoG in the nuclei of granule cells, indicating that HFD treatment increased oxidative stress in the *App^NL−F/NL−F^* hippocampus. HFD treatment also decreased the expression of TTR, a known Aβ‐binding protein, in *App^NL−F/NL−F^* mice but not wild‐type mice, suggesting that decreased levels of TTR may be a cause of the increased Aβ deposition in the hippocampus of HFD‐fed *App^NL−F/NL−F^* mice. It is likely that chronic HFD treatment increased oxidative DNA damage in the *App^NL−F/NL−F^* mouse brain, thereby aggravating the AD pathology through alteration of the gene expression.

Salas *et al*. showed that chronic HFD treatment from 2 to 18 months of age does not trigger any AD pathology in male *App^NL/NL^* mice, except for a mild impairment in both hippocampal long‐term potentiation and social memory. In contrast, we found that chronic HFD treatment from 6 to 18 months of age does aggravate hippocampal AD pathology in male *App^NL−F/NL−F^* mice, with cognitive impairment in an MWM test. In the aforementioned study, *App^NL/NL^* mice were fed an HFD containing 60% kcal from fat for 16 months. In contrast, in our study, *App^NL−F/NL−F^* mice were fed an HFD containing 40% kcal from fat for 12 months. In both cases, mice developed similar levels of metabolic syndrome or T2DM, namely increased body weight and fasting blood glucose levels, and impaired glucose tolerance. Because in comparison with *App^NL/NL^* mice, *App^NL−F/NL−F^* mice have an additional Iberian “F” mutation in the *App* gene, which is known to increase Aβ42 production by altering the γ secretase processing of the C‐terminal end of Aβ (Saito et al., 2014), our results clearly indicate that the Iberian “F” mutation is essential to make *App^NL−F/NL−F^* mice susceptible to T2DM as a risk factor for the pathogenesis of AD. *App^NL/NL^* mice mostly produce humanized Aβ40, which is detected as both soluble and insoluble forms, due to the Swedish “NL” mutation being upstream of the β secretase processing site of Aβ. However, they do not develop Aβ plaques at all (Saito et al., 2014; Salas et al., 2018). On the other hand, *App^NL−F/NL−F^* mice produce similar levels of humanized Aβ but dominantly Aβ42 (Aβ42/Aβ40 > 5), and thus develop Aβ plaques in both the cortex and hippocampus (Masuda et al., 2016; Saito et al., 2014). Taken together with the findings of previous studies, we conclude that T2DM does not trigger AD pathology but does exacerbate pre‐existing AD pathology, at least Aβ plaque formation. Moreover, our results indicate that AD pathology does not induce T2DM in RD‐fed *App^NL−F/NL−F^* mice, which had been observed in transgenic models of AD.

It has been well‐documented that an HFD or T2DM interfere with hippocampal functioning (Cordner & Tamashiro, 2015; Kanoski & Davidson, 2011), and *App^NL/NL^* mice have also been shown to exhibit mild impairment in hippocampal long‐term potentiation after chronic HFD treatment. We therefore examined HFD‐induced alterations of AD pathology in the *App^NL−F/NL−F^* hippocampus. The major alterations were markedly increased hippocampal Aβ deposition in *App^NL−F/NL−F^* mice, especially in the zone covering the upper part of the molecular layer of DG, the stratum lacunosum and stratum radiatum of CA1 (MSS), and increased microgliosis in the same zone. The affected zone contains niches for synapses from the perforant path and the Schaffer collateral pathway in the hippocampal network; thus, it is pivotal for learning and memory (Neves et al., 2008). Although the levels of SDS‐soluble Aβ remained unchanged in comparison with the RD‐fed *App^NL−F/NL−F^* hippocampus, the levels of SDS‐insoluble and FA‐extractable Aβ were increased twofold in the HFD‐fed *App^NL−F/NL−F^* hippocampus. The SDS‐soluble fraction contains Aβ derived from the membrane‐bound and cytosolic forms, while the insoluble fraction mostly contains plaque‐associated Aβ (Kawarabayashi et al., 2001). Because there was no significant change in APP processing toward Aβ formation, HFD treatment likely promoted Aβ aggregation and deposition by the suppression of its clearance. This is consistent with a recent study reporting that HFD increased SDS‐insoluble levels of Aβ in the cortex of a transgenic mouse line carrying the Swedish mutation, with a reduced Aβ clearance independent of insulin‐degrading enzyme or neprilysin (Wakabayashi et al., 2019). Although soluble prefibrillar Aβ oligomers are classified as the most toxic species (Kayed & Lasagna‐Reeves, 2013), plaques can release toxic Aβ intermediates, thus functioning as “reservoirs” of toxic oligomers (Haass & Selkoe, 2007; Thal et al., 2015). It is likely that chronic HFD treatment increases the levels of toxic Aβ in the limited zone of the hippocampus, thereby exacerbating the AD pathology, such as plaque formation and microgliosis, as observed in HFD‐fed *App^NL−F/NL−F^* mice.

Gene expression profiling by a microarray analysis revealed that HFD treatment and *App^NL−F/NL−F^* mutation have a synergetic effect to activate the microglia in the hippocampus without significant changes in the expression of marker genes for neural stem/progenitor cells, neurons, and oligodendrocytes (Table 1). CD68‐positive microglia were mainly detected in the MSS zone surrounding Aβ plaques in the HFD‐fed *App^NL−F/NL−F^* hippocampus, as reported in *App^NL−G−F/NL−G−F^* mice with another toxic Arctic “G” mutation exhibiting more severe AD pathology (Castillo et al., 2017; Saito et al., 2014). The levels of GFAP‐positive astrocytes detected in the hippocampus of wild‐type and *App^NL−F/NL−F^* mice did not differ to a statistically significant extent, with or without HFD treatment. Masuda et al. (2016) reported no changes in hippocampal GFAP levels between 18‐month‐old *App^NL−F/NL−F^* and wild‐type mice, indicating that neither *App^NL−F/NL−F^* mutation nor HFD treatment significantly affects the activation status of astrocytes in the hippocampus.

We found that HFD‐fed *App^NL−F/NL−F^* mice exhibited a significantly reduced GCL volume with much higher levels of 8‐oxoG accumulation in the nuclei of the granule cells in the GCL in comparison with RD‐fed mice. The increased accumulation of 8‐oxoG, a marker of oxidative stress, in the granule cells, and to a lesser extent in the CA1 pyramidal neurons, suggests that the production of reactive oxygen species in these neurons is enhanced by the increased toxic Aβ accumulation or by the consequence of microgliosis; thus, it may cause hippocampal atrophy without cell death by affecting dendrites stability and architecture as previously reported (Schoenfeld et al., 2017). The granule cells in the GCL or pyramidal cells in the CA1 themselves are likely to produce toxic Aβ because the MSS zone, which contains niches for synapses to their dendrites, accumulates the highest levels of Aβ. Moreover, we found that the expression of TTR in these neurons is significantly decreased in HFD‐fed *App^NL−F/NL−F^* mice in comparison with RD‐fed mice. TTR is a multifunctional protein that can bind Aβ peptide and suppress its aggregation, and which also promotes its clearance (Ghadami et al., 2020; Vieira & Saraiva, 2014). It has been shown that the hemizygous deletion of the *Ttr* gene in *APP^Swe^/PS1^deltaE9^* mice results in accelerated Aβ deposition (Choi et al., 2007), and that the overexpression of a human *TTR* transgene was ameliorative in the APP23 mice (Buxbaum et al., 2008). These results strongly suggest that the decreased expression of TTR in the hippocampal neurons of HFD‐fed *App^NL−F/NL−F^* mice is one of the direct causes of the increased Aβ deposition. We therefore speculate that decreased TTR levels may be responsible for the HFD‐induced reduction in Aβ clearance previously reported by Wakabayashi et al., 2019.

TTR is mainly expressed in the liver and choroid plexus and secreted into the blood or cerebrospinal fluid; furthermore, its levels are known to be decreased in AD patients in comparison with age‐matched controls (Velayudhan et al., 2012). Recently, it has been shown that TTR is expressed in the neurons in the cortex or hippocampus in both humans and mice (Li & Buxbaum, 2011; Oka et al., 2016), as we observed in the present study. The expression of TTR was reportedly altered in the brain of various mouse models of AD and closely associated with the level of oxidative stress (Oka et al., 2016; Sharma et al., 2019; Stein & Johnson, 2002). 8‐OxoG, or its repair reaction, is known to cause epigenetic alterations in the gene expression under oxidative conditions (Ba & Boldogh, 2018), suggesting a functional contribution of 8‐oxoG to the altered expression of TTR in the hippocampal neurons.

We recently demonstrated that the increased accumulation of 8‐oxoG in the granule cells in the GCL impairs hippocampal neurogenesis, thus inducing hippocampal atrophy and mild cognitive impairment in aged female mice (Haruyama et al., 2019). In the present study, we only examined male mice in order to avoid the strong effects of sex hormones or the estrus cycle; however, it is likely that female *App^NL−F/NL−F^* mice may exhibit a much higher susceptibility to HFD; thus, the AD pathology would be more strongly exacerbated because sex‐dependent TTR modulation of brain Aβ levels or adult neurogenesis has been reported (Oliveira et al., 2011; Vancamp et al., 2019). Further studies are necessary to better understand the mechanism by which HFD treatment causes alterations in the gene expression, production, or clearance of the Aβ peptide, oxidative stress, and inflammatory responses. In particular, answering the question as to how T2DM affects the interaction of TTR and Aβ in the AD brain will help to establish new strategies for AD treatment.

## 4 EXPERIMENTAL PROCEDURES

### 4.1 Experimental animals

Heterozygous *App^+/NL−F^* mice carrying a humanized Aβ sequence (G676R, F681Y, R684H), Swedish (KM670/671NL), and Beyreuther/Iberian (I716F) mutations were previously established (Saito et al., 2014) and backcrossed onto the C57BL/6J background for 9 generations. Homozygous *App^NL−F/NL−F^* and wild‐type mice were obtained by mating between heterozygous mice and were maintained as inbred lines. Mice were maintained in an air‐conditioned specific‐pathogen‐free room at 22°C with a 12:12‐h light and dark cycle (lights on 08:00, off at 20:00), with *ad libitum* access to food and water. The handling and killing of animals were carried out in accordance with the national prescribed guidelines. Ethical approval for the study was granted by the Animal Care and Use Committee of Kyushu University, Fukuoka, Japan.

### 4.2 HFD treatment and metabolic assessment

To avoid strong effects of sex hormones or the estrus cycle in female mice, we used male *App^NL−F/NL−F^* and wild‐type mice. At 6 months of age (25 weeks), mice were individually housed and fed *ad libitum* with either an RD (CA‐1, 13.8% of total calories from fat, Clea Japan Inc., Tokyo, Japan) or HFD (custom diet, 40% of total calories from fat and 0.15% from cholesterol, Oriental Yeast Co., Tokyo, Japan) until the end of the experiment (18 months of age). Using an animal balance (DH‐R610N, Shinko Denshi Co., Ltd.), the body weight of all mice was measured and recorded once a week from 25 to 50 weeks of age and then at 58 and 78 weeks of age. The weekly intakes of food and water were calculated by subtracting the remaining amounts of food and water at the end of the week from the amount fed to each mouse at the beginning of the week. Every other week, blood samples were collected from tail after 6 h fasting, and the fasting blood glucose level was measured using a Freestyle Flash glucometer (NIPRO Co., Ltd, Osaka, Japan). At 18 months of age, mice were subjected to an intraperitoneal glucose (2 g/kg) tolerance test (IPGTT). Briefly, mice were fasted for 6 h before the IPGTT and blood samples were collected from the tail at the following time points: before glucose injection (0 min), and 30, 60, 90, and 120 min after glucose injection.

### 4.3 Morris water maze test

A Morris water maze test was performed at 18 months of age in all groups, as described previously (Haruyama et al., 2019) with some modifications. Detailed procedures can be found online in the Appendix S1.

### 4.4 Brain sample preparation

After the final behavioral test, mice were sacrificed, and their brains were dissected as previously described (Oka et al., 2016). For protein preparation, frozen mouse hippocampi were homogenized in 2× sodium dodecyl sulfate (SDS) sample buffer (125 mM Tris‐HCl, pH 6.8, 4% SDS, 10% glycerol) with 1% protease inhibitor cocktail and 1% phosphatase inhibitor cocktail (Nacalai Tesque, Inc., Kyoto, Japan). After sonication, the homogenates were centrifuged at 100,000 × *g* for 30 min at 20°C using an Optima TLX ultracentrifuge and a TLA55 rotor (Beckman, Palo Alto, CA, USA); the supernatant was stored as soluble protein (SDS soluble) fraction. The pellet was subsequently washed and centrifuged again and finally dissolved in 70% formic acid (FA). After all the formic acid had been removed by freeze‐drying, protein was dissolved in DMSO, diluted with the same volume of 4×SDS sampling buffer, and stored as the insoluble (FA‐extractable) fraction. Protein concentrations were measured using an XL‐Bradford Protein Assay Reagent Kit (Antegral Co., Ltd., Tokyo, Japan).

### 4.5 Dot blotting of soluble and insoluble Aβ

Dot blotting was performed using a dot blotting assay system (Bio‐Dot® Microfiltration Apparatus, Bio‐Rad Laboratories, Hercules, CA, USA). The procedure was performed according to the manufacturer's guidelines. For each sample, 2.0 μg of SDS soluble or 2 µl of formic acid‐soluble protein diluted in 100 μl of TBS was applied to a PVDF membrane in triplicate. After blotting, the membrane was detached from the apparatus and blocked with 5% skim milk in TBS +0.1% Tween 20 (TBST) for 1 h at room temperature, followed by incubation with mouse anti‐human Aβ 82E1 antibody, which recognizes the N‐terminal end of humanized Aβ, overnight at 4°C. The next day, the membrane was washed and incubated with appropriate HRP‐conjugated secondary antibodies for 1 h at room temperature. The blot was TBST, incubated in luminol HRP substrate (EZWestLumi plus, ATTO, Tokyo, Japan), and imaged on an EZ capture MG (ATTO). The intensity of the dots was measured using ImageJ 1.52 (NIH, Bethesda, MD, USA).

### 4.6 Western blotting

Western blotting was performed to measure the levels of different proteins in the hippocampus. Detailed procedures can be found online in the Appendix S1.

### 4.7 Immunofluorescence microscopy and histochemical analyses

Immunofluorescence microscopy and histochemical analyses were performed as previously described (Castillo et al., 2017; Haruyama et al., 2019). Detailed procedures can be found online in the Appendix S1.

### 4.8 RNA isolation and the microarray analyses

Preparation of RNA and the microarray analysis were performed as previously described (Castillo et al., 2017). Total RNA extracted from frozen hippocampi using Isogen (Nippon Gene, Tokyo, Japan) was subjected to microarray analysis with an Affymetrix Mouse Gene 2.0 ST Array. The generated CEL files were imported into the Transcriptome Analysis Console 4.0 software program (Affymetrix), and gene‐level estimates were obtained for all transcript clusters. All microarray data were deposited in the GEO database (accession number GSE152539).

### 4.9 Statistical analyses

All statistical analyses were performed using JMP Pro 14.2.0 (SAS Institute Japan Ltd., Tokyo, Japan). All *t* tests were two‐tailed. A multivariate analysis of variance (MANOVA) and two‐way ANOVA were used to assess the interaction between factors. When significant interactions were detected, a post hoc Tukey HSD test was used to adjust for multiple comparisons. *p* values of <0.05 were considered to indicate statistical significance.

## CONFLICT OF INTEREST

None declared.

## AUTHOR CONTRIBUTIONS

R.I and N.H performed animal experiments. N.A performed the RNA preparation and microarray. N.A and G.M. performed the microarray data analysis. G.M performed sample preparation, immunostaining and immunofluorescence microscopy, and quantification. G.M performed protein blotting and quantification. K.S. assisted in designing the experiments. T.S. and T.S. provided the *App^+/NL−F^* mice. R.I. and Y.N. designed the animal experiments. Y.N., G.M., and N.A. designed the gene expression profiling and pathological experiments, prepared the figures, and conducted statistical analyses. G. M. and Y.N. wrote the paper. All authors discussed the data obtained and contributed to the preparation of the manuscript.

## Supporting information

App S1

Tab S1

Tab S2

Tab S3

## Data Availability

Originals of all datatypes are available on request to the corresponding author. Microarray data were deposited in the NCBI Gene Expression Omnibus GEO database under the accession number GSE152539.
