# Supplementary material for: A high‐fat diet exacerbates the Alzheimer's disease pathology in the hippocampus of the App NL−F/NL−F knock‐in mouse model
Source: Aging Cell. 2021 Jul 10;20(8):e13429. doi: 10.1111/acel.13429 (PMC8373331; doi:10.1111/acel.13429)
Supplement: Supplementary file 1 — App S1 [file ACEL-20-e13429-s004.pdf]

SUPPORTING INFORMATION

A high-fat diet exacerbates the Alzheimer's disease pathology in the hippocampus of the *App*$^{NL\text{-}F/NL\text{-}F}$ knock-in mouse model

Running title: High-fat diet treatment of *App*$^{NL\text{-}F/NL\text{-}F}$ mice

Guianfranco Mazzei[1], Ryohei Ikegami[1], Nona Abolhassani[1], Naoki Haruyama[1], Kunihiko Sakumi[1], Takashi Saito[2,3], Takaomi C. Saido[2], Yusaku Nakabeppu[1]

[1]Division of Neurofunctional Genomics, Department of Immunobiology and Neuroscience, Medical Institute of Bioregulation, Kyushu University, Fukuoka, 812-8582, Japan.

[2]Laboratory for Proteolytic Neuroscience, RIKEN Center for Brain Science, Saitama, Japan.

[3]Department of Neurocognitive Science, Institute of Brain Science, Nagoya City University Graduate School of Medical Sciences, Nagoya, Japan.

Correspondence

Nona Abolhassani and Yusaku Nakabeppu, Division of Neurofunctional Genomics, Department of Immunobiology and Neuroscience, Medical Institute of Bioregulation, Kyushu University, Fukuoka, 812-8582, Japan.

E-mail: nona_ab@bioreg.kyushu-u.ac.jp (NA), yusaku@bioreg.kyushu-u.c.jp (YN)

This file contains Supplementary Experimental Procedures, References, and Figures.

1. | SUPPLEMENTARY EXPERIMENTAL PROCEDURES

1.1 | Experimental Animals

The homozygous triple-transgenic mouse model of AD (3xTg-AD-H), carrying a homozygous *Psen1*^{M146V} knock-in mutation and the homozygous mutant transgenes for *APP*^{Swe} and *MAPT*^{P301L}, were previously established (Oddo et al., 2003), and maintained (Oka et al., 2016).

1.2 | Genotyping of animals used in this study

Genotyping of the *App*$^{NL\text{-}F}$ allele was performed by genomic polymerase chain reaction (PCR) (forward primers, E16WT: 5'-ATCTCGGAAGTGAAGATG-3', E16MT: 5'-ATCTCGGAAGTGAATCTA-3'; reverse primers, WT: 5'-TGTAGATGAGAACTTAAC-3', loxP : 5'-CGTATAATGTATGCTATACGAAG -3') as previously described by Saito et al. 2014.

1.3 | Morris water maze test

A Morris water maze test was performed at 18 months of age in all groups, as described previously (Haruyama et al., 2019) with some modifications. Mice were moved to the behavioural analysis room at least one week before the start of the test. The test was performed in a controlled room with a temperature of 25°C. The water maze consisted of a circular pool (diameter, 100 cm; depth, 30 cm) filled to 15 cm with room-temperature tap water (23°C). Several visual cues were placed on the interior of the pool. Performance was scored using a video tracking system, WaterMaze (Actimetrics Inc., Wilmette, IL, USA). To evaluate spatial working memory, mice were trained to escape onto a circular, clear, Plexiglas platform of 15 cm in diameter that was submerged 0.5 cm beneath the surface of the water, and which was invisible to the mice while swimming. Mice were given four consecutive trials per day for 11 consecutive days. When mice did not reach the platform within 60 s in a trial, they were guided to the platform and allowed to stay there for 15 s. To evaluate memory retrieval, a probe test was conducted in the pool without the platform for 60 s, 24 h after the last training acquisition trial.

1.4 | Brain tissue preparation

After the final behavioral test, mice were anaesthetized with a combination of medetomidine (0.3 mg/kg), midazolam (4.0 mg/kg), and butorphanol (5.0 mg/kg), then perfused transcardially with 20 mL of saline solution. For protein/RNA preparations, hippocampi were quickly dissected, frozen in liquid nitrogen, and stored at –80 °C until further extraction. For the immunohistochemical analysis, mice were further perfused with 1 mL/g of 4% paraformaldehyde in 1 × phosphate-buffered saline (PBS). The brain was removed and post-fixed with 4% paraformaldehyde at 4 °C for 24 h, then cryoprotected in 20% and 30% sucrose in 1 × PBS at 4 °C for 24 h each. Coronal brain blocks were mounted in FSC 22 Frozen Section Media (Leica Microsystems K.K., Tokyo, Japan), frozen in liquid nitrogen and stored until use at −80 °C. Brain blocks were cut on a cryostat at a thickness of 40 μm and collected as free-floating sections in PBS.

1.5 | List of antibodies used in the study

Antibody	Abbreviation used in the manuscript	Clone	Brand	Catalogue number
anti-phospho-IRS1(Tyr608)	pIRS1^{Y608}	-	Merck	09-432
anti-phospho-IRS1(Ser636/639)	pIRS1$^{S632/635*}$	-	Cell Signaling	#2388S
anti-IRS1	IRS-1	-	Cell Signaling	#2382S
anti-Insulin receptor β	INSRβ	CT-3	Santa Cruz	sc-57342
anti-phospho-AKT(Ser473)	pAKT^{S473}	-	Cell Signaling	#9271S
anti-AKT	AKT	-	Cell Signaling	#9272S
anti-PSD95	PSD95	D27E11	Cell Signaling	#3450S
anti-Synaptophysin	Syn	-	Sigma	SAB4502906-100UG
Anti-Human Amyloidβ(N)	82E1	82E1	IBL	10323
anti-mouse CD68	CD68	FA-11	Biorad	MCA1957GA
anti-IL-1β	IL-1β	H-153	Santa Cruz	sc-7884
anti-8-OHdG	8-oxoG	N45.1	JaICA	MOG-100P
anti-Prealbumin	TTR	E-1	Santa Cruz	sc-377517
anti-Prealbumin	TTR	-	Dako	A0002-02
anti- Phospho-Tau (Ser202, Thr205)	AT8	AT8	Thermo-Fisher	MN1020
anti-APP A4, (a.a. 66-81)	22C11	22C11	Merck	MAB348
anti-β-Amyloid, 1-16	6E10	6E10	Biolegend	803002
anti-APP, C-Terminal	APP-CT	-	Sigma	A8717
anti-GFAP	GFAP	-	Biolegend	840001
anti-4Hydroxynonenal	4-HNE	HNEJ-2	abcam	ab48506

*Recognizes mouse IRS phosphorylated at Ser 632/635

1.6 | Western blotting

For the detection of APP, APP-derived fragments, PSD95, synaptophysin, IL-1β, and 4-HNE-modified proteins, 20 µg of SDS soluble protein homogenates were separated by bis Tris SDS-PAGE using MES SDS Running Buffer (NP0002; Thermo Fisher Scientific K.K, Tokyo, Japan) and transferred onto 0.2-µm nitrocellulose membrane (10600001; Global Life Sciences Solutions USA LLC, Marlborough, MA, USA). For Aβ detection, the membrane was subsequently incubated in 1 × PBS at 95 °C for 5 min, as previously described (Noguchi et al. 2009). Next, membranes were blocked for 1 h at room temperature in TBST containing appropriate blocking reagents (see table below). Membranes were then incubated with the corresponding primary antibodies overnight at 4 °C with gentle shaking. The following day, the membranes were washed with TBST and incubated in solutions containing the

corresponding HRP-linked secondary antibody (7076S or 7074S, 1:4000; Cell Signaling Technology, Inc., Danvers, MA, USA) for 1 h at room temperature. The blot was then washed 3 times for 10 min with TBST, incubated in luminol HRP substrate (Western BLoT HRP Substrate; Takara Bio Inc., Kusatsu, Japan), and its image was captured by an EZ capture MG (ATTO). The intensity and background of the bands was measured using the ImageJ software program, 1.52 (NIH, Bethesda, MD, USA). For the detection of insulin signaling proteins and transthyretin, 20 µg of SDS soluble protein homogenate was electrophoresed by SDS-PAGE and blotted onto a 0.45-µm PVDF membrane and processed following the procedures described above.

Antibody	Dilution	Blocking solution	Antibody diluted in
anti-phospho-IRS1(Tyr608)	1:1000	Blocking One-P	same as blocking
anti-phospho-IRS1(Ser636/639)	1:1000	Blocking One-P	same as blocking
anti-IRS1	1:1000	1% milk	same as blocking
anti-INSRβ (CT-3)	1:1000	1% milk	same as blocking
anti-phospho-AKT(Ser473)	1:1000	1% milk	same as blocking
anti-AKT	1:1000	5% BSA	1% BSA
anti-PSD95 (D27E11)	1:1000	2.5% milk	1% milk
anti-Synaptophysin	1:1000	2.5% milk	1% milk
anti-IL-1β (H-153)	1:1000	2.5% milk	1% milk
anti-Prealbumin (E-1)	1:100	5% milk	1% milk
anti-APP A4 (22C11)	1:100	1% milk	same as blocking
anti-Aβ 1-16 (6E10)	1:2000	5% milk	1% milk
Anti-FL-AAP CT	1:4000	1% milk	same as blocking
anti-4-HNE (HNEJ-2)	1:100	5% milk	1% milk

1. 7 Immunofluorescence microscopy and histochemical analysis

For immunofluorescence microscopy, sections were blocked in 2× Block Ace solution (Dainippon Pharmaceutical, Osaka, Japan) for 2 h at room temperature, then incubated with the corresponding primary antibody mix (see table below) overnight at 4 °C. For the immunodetection of transthyretin, sections were previously incubated in 10% formalin solution for 15 min at room temperature. The next day, sections were incubated in the corresponding Alexa Fluor-labelled secondary antibodies (Thermo Fisher Scientific K.K, Tokyo, Japan) for 45 minutes at room temperature, followed by 0.05 µg/ml DAPI for 10 min at room temperature. Fluoro-JadeC (AG325; Merck KGaA, Darmstadt, Germany) staining was performed according to the manufacturer's instructions. Finally, sections were mounted on glass slides using

VECTASHIELD Mounting Medium (Vector Laboratories, Ltd., Burlingame, CA, USA). Cresyl violet staining was used to stain neural cells located in the dentate gyrus. For immunofluorescence images, tile scan images of the whole hemisphere, or high magnification z-stacks, were obtained using a laser scanning confocal microscope system (LSM700, Carl Zeiss Microscopy, Tokyo, Japan) with the Zen 2012 software program (Carl Zeiss Microscopy). Cresyl violet staining images were acquired with a Nikon Eclipse 80i microscope equipped with a Virtual slice module (Stereo Investigator, MBF Bioscience, Williston, VT, USA). Volume estimations were performed using the Cavalieri method, as previously described by Howard & Reed (2004) using 5 serial sections (every 6th slice) in total. For immunofluorescence quantification, at least 4 serial sections (every 6th slice) were quantified per mouse brain. The quantification of all images was performed using ImageJ 1.52 (NIH, Bethesda, MD, USA).

Antibody	Dilution	Blocking solution	Antibody diluted in
Anti-Human Amyloidβ (N)	1:2000	2× Block Ace	1/10 × Block Ace
anti-mouse CD68	1:500	1× Block Ace	1/10 × Block Ace
anti-8-OHdG	1:80	1× Block Ace	1/10 × Block Ace
anti-Prealbumin (E-1)	1:100	1× Block Ace	1/10 × Block Ace
anti-Prealbumin	1:100	1× Block Ace	1/10 × Block Ace
anti-GFAP	1:2000	1× Block Ace	1/10 × Block Ace

1.8 | Quantitative immunodetection of 8-oxoguanine in DNA

For the immunodetection of 8-oxoG, free-floating sections were processed as previously described by Haruyama et al. 2019. To detect 8-oxoG in nuclear DNA, sections were pre-treated with RNase (5 mg/ml; Sigma-Aldrich Japan, Tokyo, Japan) and then were further pre-treated with 2 N HCl to denature the nuclear DNA. Subsequently, sections were processed for immunohistochemistry with an anti-8-oxo-dG primary antibody (clone N45.1, 1:80, Japan Institute for the Control of Aging, Nikken Seil Co., Ltd., Fukuroi, Shizuoka, Japan) then processed using a Vector ABC kit (Vector Laboratories) with the proper biotinylated secondary antibody. The 3,3′-diaminobenzidine (DAB)/nickel (Vector Laboratories) reaction was then used to visualize the bound secondary antibody. Images were acquired with a Nikon Eclipse 80i microscope equipped with a Virtual slice module (Stereo Investigator, MBF Bioscience). Digital images of 8-oxoG were converted to grayscale, then the signal intensity of 8-oxoG

immunoreactivity was quantified using ImageJ 1.52 (NIH, Bethesda, MD, USA). The region of interest was defined manually, and the mean pixel intensities were determined after subtracting the background intensity calculated for each experiment. At least 4 serial sections (every 6th slice) were quantified per mouse brain.

1.9 | Immunohistochemical detection of phosphorylated Tau

Serial coronal sections of 40 µm in thickness were prepared using a cryostat and were collected as free-floating sections. The sections were washed in PBD (0.3% Triton X-100 in PBS) 3 times for 5 min. Then blocked in Block Ace® solution (Dainippon Pharmaceutical, Osaka, Japan) for 30 mins, followed by incubation overnight at 4 °C with a mouse monoclonal antibody against phosphorylated Tau at Ser202/Thr205 (Clone AT8, MN1020, 1:200, Thermo Fisher Scientific K.K, Tokyo, Japan) diluted in 10% Block Ace. The next day, the sections were immersed in a solution of 1% hydrogen peroxide in methanol/PBD (1:1) for 10 min, to block endogenous peroxidase activity. Sections were then processed using a Vector ABC kit (Vector Laboratories, Ltd., Burlingame, CA, USA) with an appropriate biotinylated secondary antibody, and the peroxidase reaction product was detected using a 3,3´diaminobenzidine/nickel reaction (Vector Laboratories). Images of the entire coronal sections were obtained using a Nikon Eclipse 80i microscope with a Virtual slice module in the Stereo Investigator software program (MBF Bioscience).

2. | REFERENCES

Haruyama, N., Sakumi, K., Katogi, A., Tsuchimoto, D., De Luca, G., Bignami, M., & Nakabeppu, Y. (2019). 8-Oxoguanine accumulation in aged female brain impairs neurogenesis in the dentate gyrus and major island of Calleja, causing sexually dimorphic phenotypes. *Progress in Neurobiology*, *180*, 101613. https://doi.org/10.1016/j.pneurobio.2019.04.002

Howard, V., & Reed, M. (2004). *Unbiased Stereology: Three-Dimensional Measurement in Microscopy*. CRC Press. https://books.google.co.jp/books?id=Pqh5AgAAQBAJ

Noguchi, A., Matsumura, S., Dezawa, M., Tada, M., Yanazawa, M., Ito, A., Akioka, M., Kikuchi, S., Sato, M., Noda, M., Fukunari, A., Muramatsu, S. I., Itokazu, Y., Sato, K., Takahashi, H., Teplow, D. B., Nabeshima, Y. I., Kakita, A., Imahori, K., & Hoshi, M. (2009). Isolation and characterization of patient-derived, toxic, high mass Amyloid β-protein (Aβ) assembly from Alzheimer disease brains. *Journal of Biological Chemistry*, *284*, 32895–32905. https://doi.org/10.1074/jbc.M109.000208

Oddo, S., Caccamo, A., Shepherd, J. D., Murphy, M. P., Golde, T. E., Kayed, R., Metherate, R., Mattson, M. P., Akbari, Y., & LaFerla, F. M. (2003). Triple-transgenic model of Alzheimer's Disease with plaques and tangles: Intracellular Aβ and synaptic dysfunction. *Neuron*, *39*, 409–421. https://doi.org/10.1016/S0896-6273(03)00434-3

Oka, S., Leon, J., Sakumi, K., Ide, T., Kang, D., LaFerla, F. M., & Nakabeppu, Y. (2016). Human mitochondrial transcriptional factor A breaks the mitochondria-mediated vicious cycle in Alzheimer's disease. *Scientific Reports*, *6*, 37889. https://doi.org/10.1038/srep37889

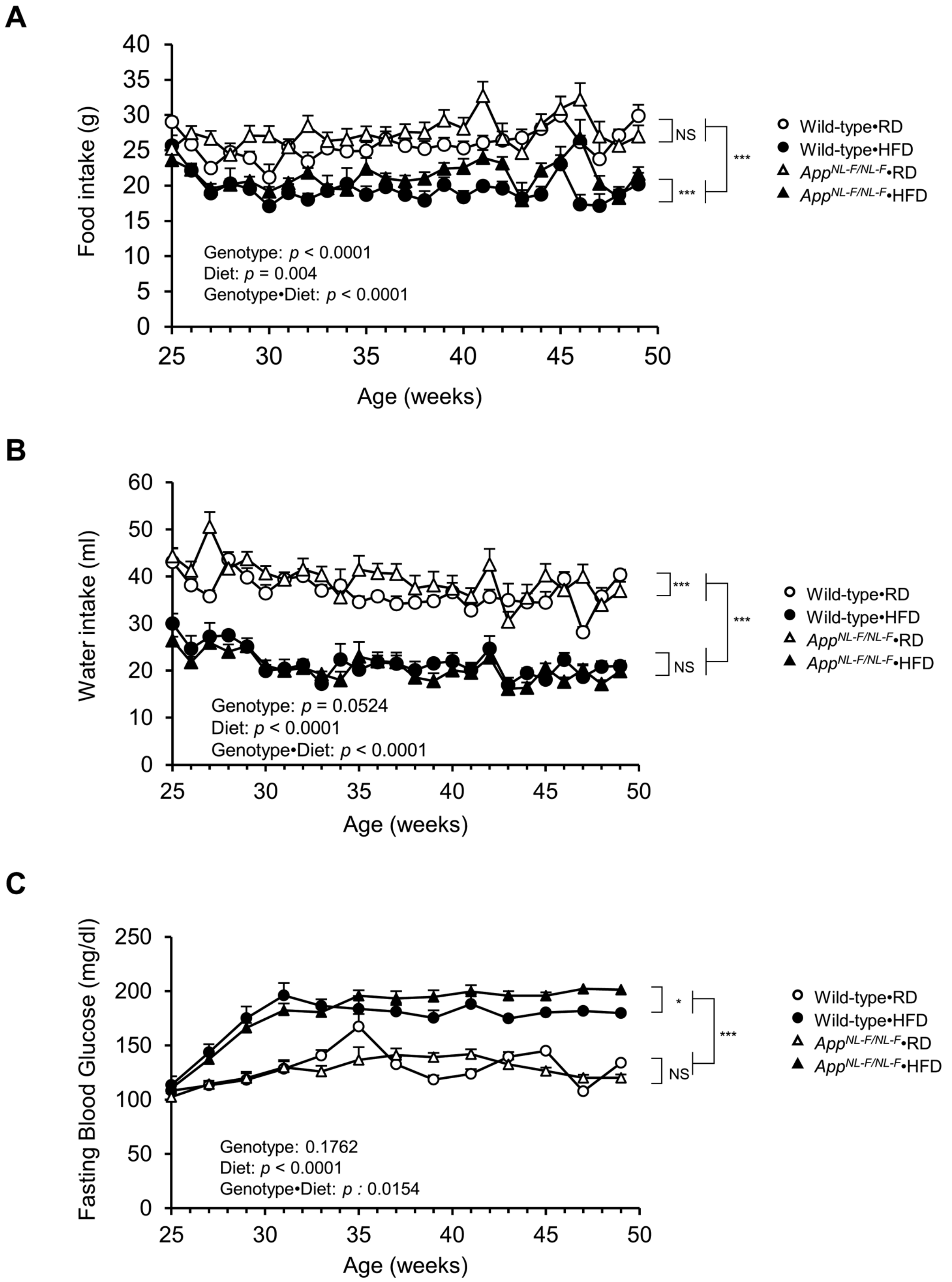

Supplementary Figure S1. Levels of food intake, water intake, and fasting glucose in *App*$^{NL\text{-}F/NL\text{-}F}$ and wild-type mice fed an RD or HFD. A. Food intake was recorded weekly. B. Water intake was recorded weekly. C. Fasting blood glucose. The blood glucose level was measured every two weeks after fasting for six hours. Data are expressed as the mean ± SEM, n = 7–13 for all experiments. The results were statistically analyzed by a two-way repeated-measures ANOVA followed by a post-hoc Tukey's HSD test, $^{*}p < 0.05$, $^{***}p < 0.001$.

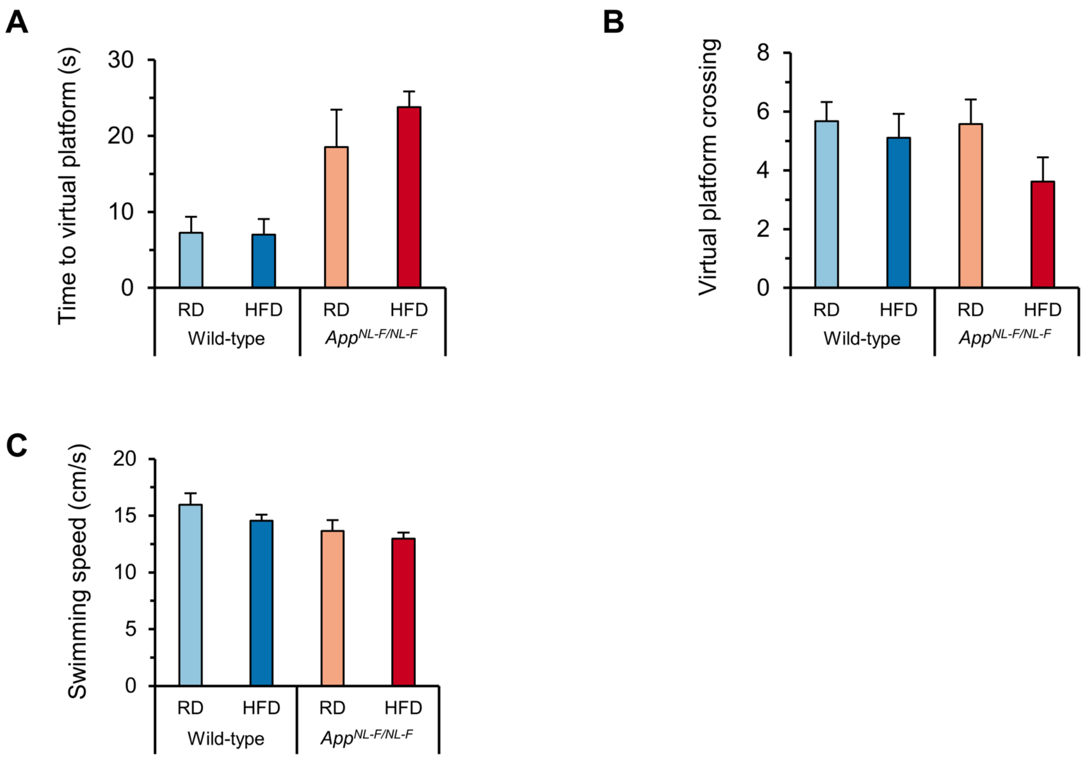

Supplementary Figure S2. Other parameters in the Morris Water Maze probe test 24 h after the last training. (A-C) A probe trial with the platform removed performed 24 h after the completion of training showed no significant difference in the time to the virtual platform, frequency of virtual platform crossing, and swimming speed among the groups. Data are expressed as the mean ± SEM, n = 11 – 14 for all groups. Statistical analyses for (A-C) were performed by a two-way ANOVA.

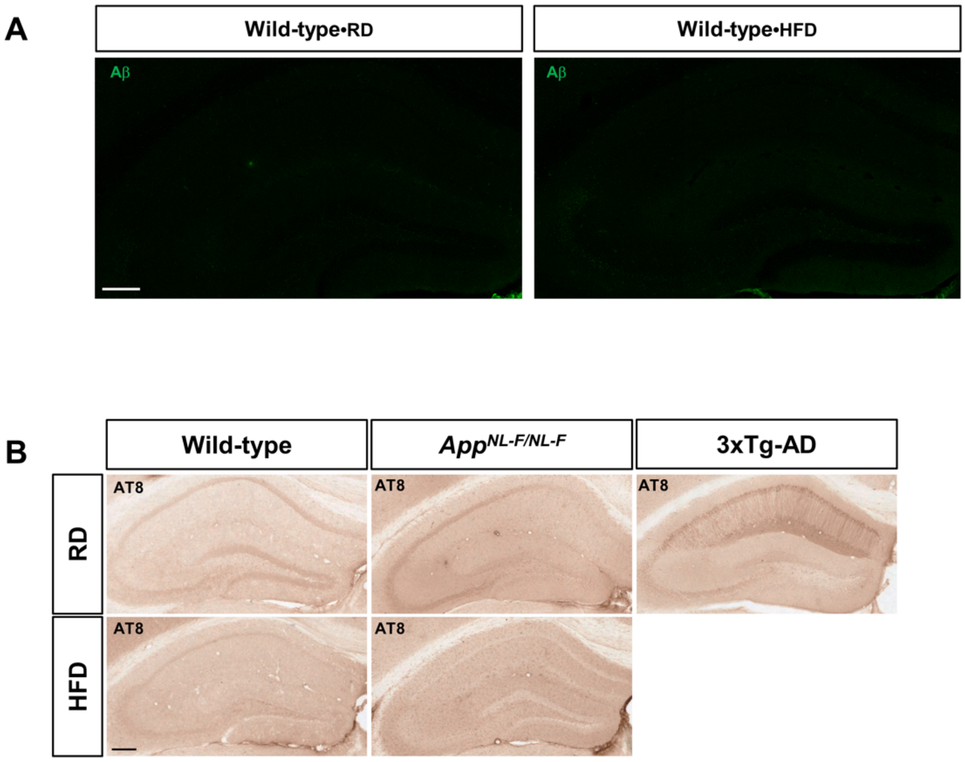

Supplementary Figure S3. Immunodetection of Aβ and Tau in the hippocampus of *App*$^{NL-F/NL-F}$ and wild-type mice. (A) Immunofluorescence detection of Aβ. Anti-Aβ 82E1 antibody showed no positive signal in the wild-type brain fed an RD or HFD. Scale bar = 200 µm. (B) Representative images of the immunohistochemical detection of Tau in the hippocampus of *App*$^{NL-F/NL-F}$ and wild-type mice fed an RD or HFD showed no positive signal. A positive control (Triple transgenic [3xTg-AD] mouse carrying a transgene of MAPTP301L, which induces Tau hyperphosphorylation) was used. Scale bar = 200 µm.

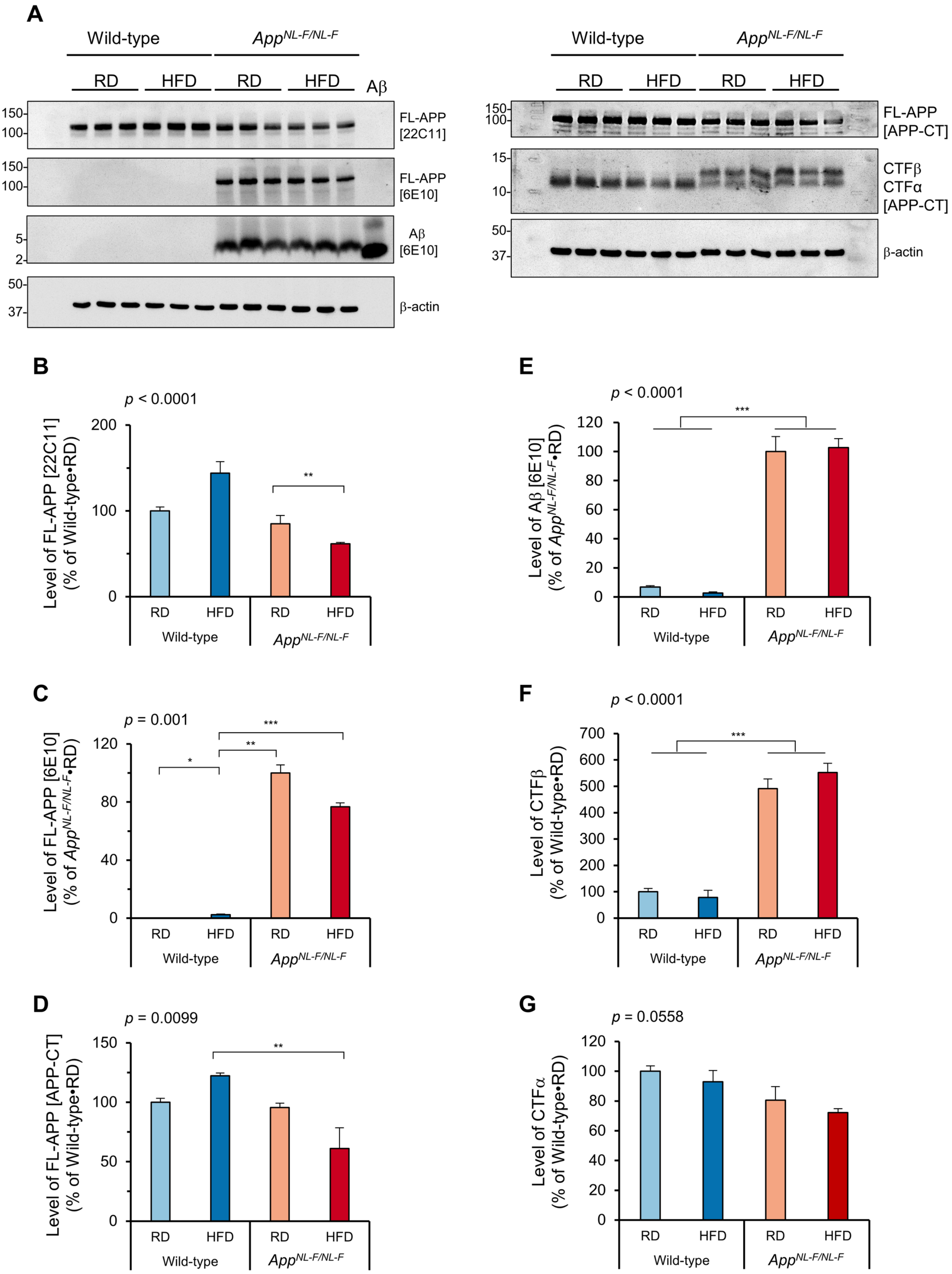
A
Wild-type
App^NL-F/NL-F
RD
HFD
Aβ
150
100
FL-APP [22C11]
FL-APP [6E10]
5
2
Aβ [6E10]
50
37
β-actin
FL-APP [APP-CT]
15
10
CTFβ
CTFα
[APP-CT]
B
p < 0.0001
Level of FL-APP [22C11] (% of Wild-type•RD)
200
0
**
E
p < 0.0001
Level of Aβ [6E10] (% of App^NL-F/NL-F•RD)
120
80
60
40
20
***
C
p = 0.001
Level of FL-APP [6E10] (% of App^NL-F/NL-F•RD)
*
F
Level of CTFβ (% of Wild-type•RD)
700
600
500
400
300
200
D
p = 0.0099
Level of FL-APP [APP-CT] (% of Wild-type•RD)
G
p = 0.0558
Level of CTFα (% of Wild-type•RD)

Supplementary Figure S4. APP processing is not affected by HFD in *App*$^{NL\text{-}F/NL\text{-}F}$ and wild-type mice. (A) Western blot analyses of full-length APP (FL-APP) and APP-derived fragments in wild-type and *App*$^{NL\text{-}F/NL\text{-}F}$ mice using antibodies to the N-terminus of APP [22C11], to the humanized Aβ [6E10], and to the C-terminus of APP [APP-CT]. (B-G) Quantification of FL-APP and APP-derived fragments in blots using β-actin as a loading control. The bar graphs show the protein/β-actin ratio relative to RD-fed wild-type mice (B, D, F, G), or RD-fed *App*$^{NL\text{-}F/NL\text{-}F}$ when 6E10 antibody to a humanized Aβ epitope was used (C, E). Data are expressed as the mean ± SEM. n = 3 for all groups. The results were statistically analyzed by a two-way ANOVA (p values are shown for each analysis) followed by post-hoc Tukey's HSD test, where $^{*}p < 0.05$, $^{**}p < 0.01$, $^{***}p < 0.001$. The levels of FL-APP in wild-type mice tend to be increased by HFD, while to be decreased in *App*$^{NL\text{-}F/NL\text{-}F}$ mice by HFD (B-D). *App*$^{NL\text{-}F/NL\text{-}F}$ mice showed significantly higher levels of Aβ and CTFβ than the wild-type mice, changes that were not altered by an HFD (E, F). There was no significant difference in the CTFα levels among wild-type and *App*$^{NL\text{-}F/NL\text{-}F}$ mice fed an RD or HFD (G).

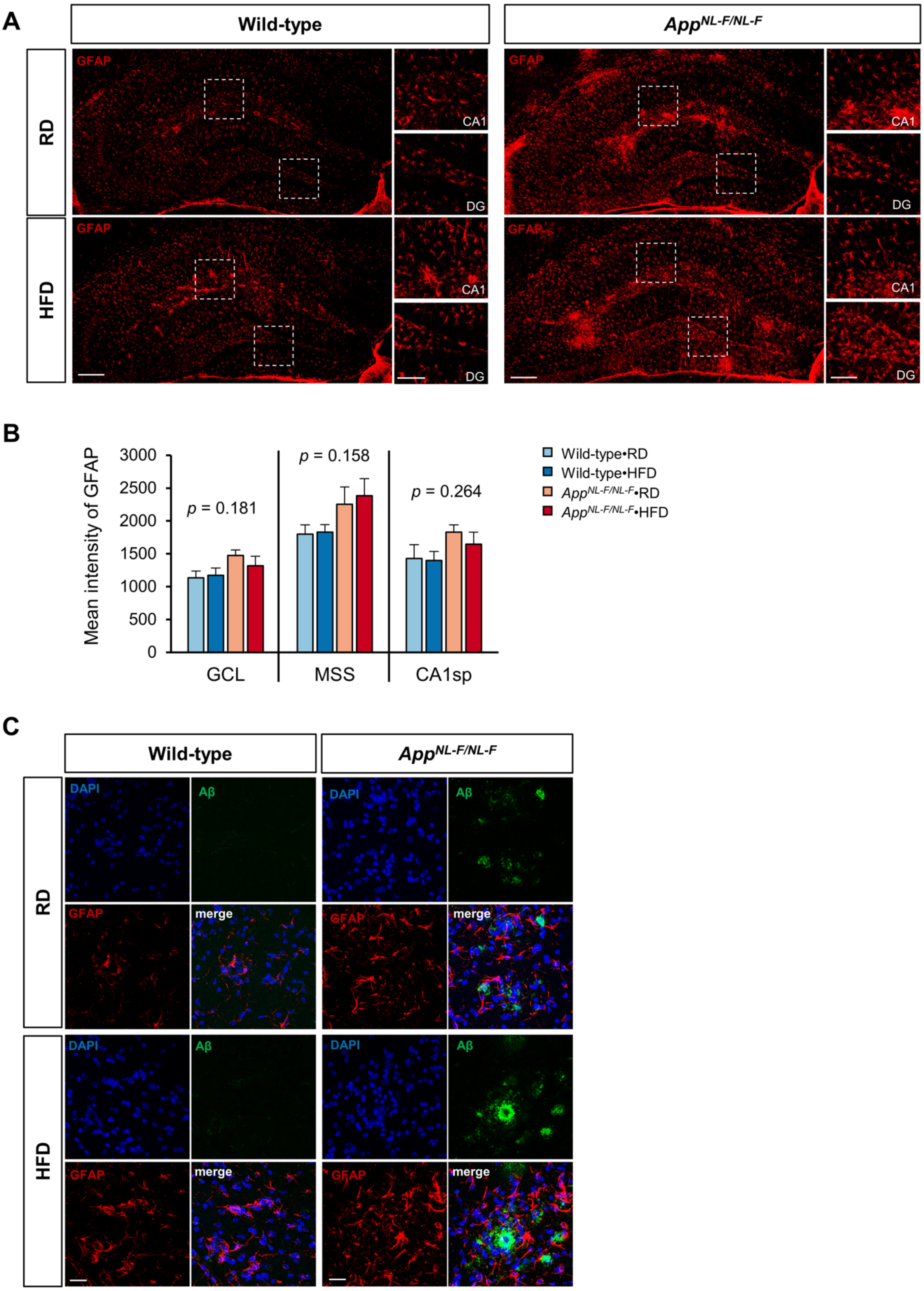
A
Wild-type
App^NL-F/NL-F
RD
HFD
GFAP
CA1
DG
B
Mean intensity of GFAP
3000
2500
2000
1500
1000
500
0
p = 0.181
p = 0.158
p = 0.264
GCL
MSS
CA1sp
Wild-type•RD
Wild-type•HFD
App^NL-F/NL-F•RD
App^NL-F/NL-F•HFD
C
Wild-type
App^NL-F/NL-F
RD
HFD
DAPI
Aβ
GFAP
merge

Supplementary Figure S5. A high-fat diet did not alter the activation of astrocytes in the hippocampus of *App*$^{NL\text{-}F/NL\text{-}F}$ mice. (A) Representative immunofluorescence images of GFAP-positive astrocytes in the hippocampus of *App*$^{NL\text{-}F/NL\text{-}F}$ and wild-type mice fed an RD or HFD. Scale bar = 200 µm (for full image) and 100 µm (for augmented areas), DG = dentate gyrus. (B) Quantification of GFAP intensity in three different zones of the hippocampus shows no marked difference in the expression of GFAP in *App*$^{NL\text{-}F/NL\text{-}F}$ mice fed an RD or HFD. (C) Double-immunofluorescence microscopy for Aβ and GFAP shows that GFAP-positive astrocytes were mainly localized around the Aβ plaques. Scale bar = 50 µm. Data are expressed as the mean ± SEM, n = 4, and four brain slices per mouse were examined. The results were statistically analyzed by a two-way ANOVA (p values for each analysis shown).

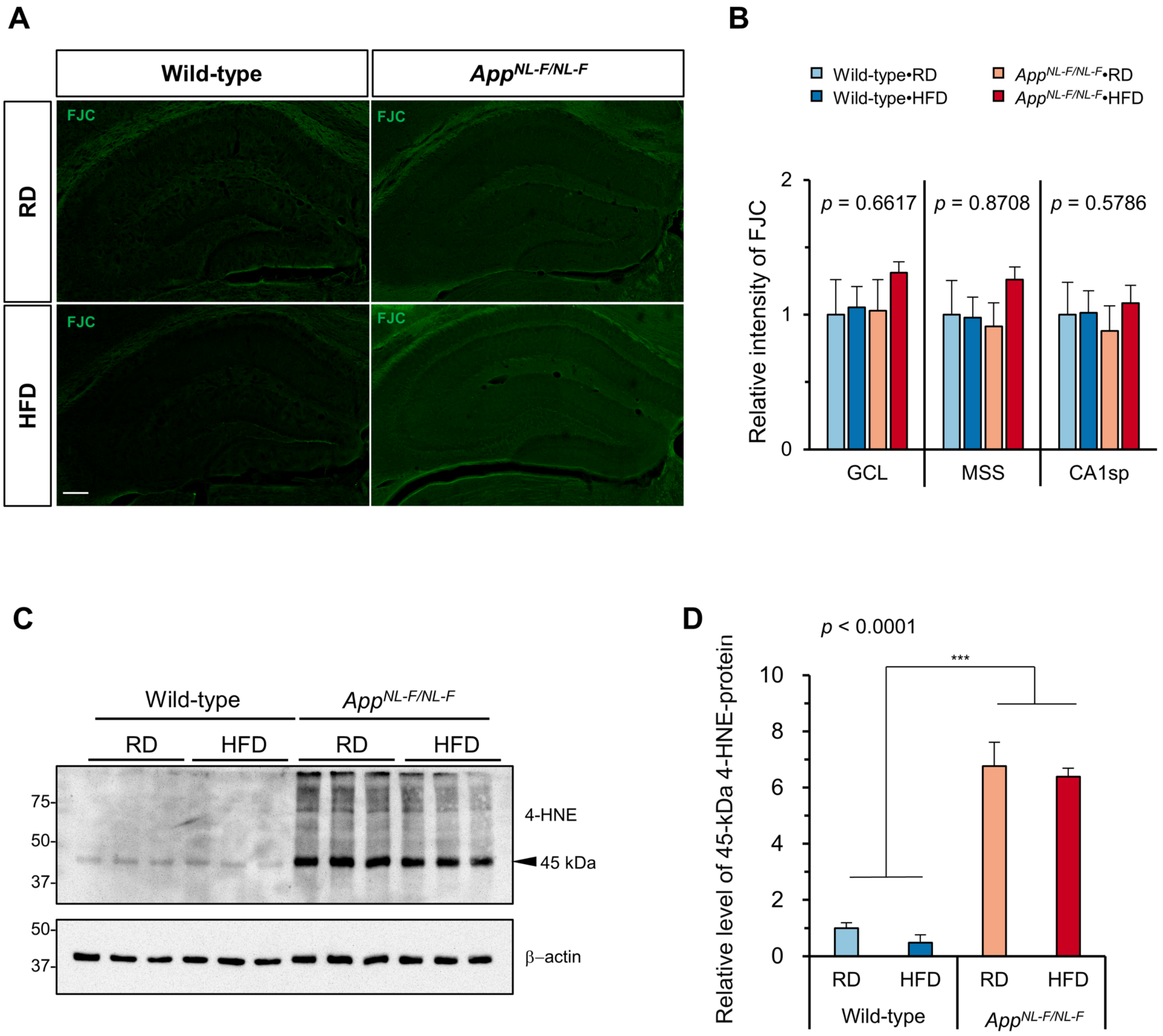
A
Wild-type
App^NL-F/NL-F
RD
HFD
FJC
B
Wild-type•RD
Wild-type•HFD
App^NL-F/NL-F•RD
App^NL-F/NL-F•HFD
Relative intensity of FJC
p = 0.6617
p = 0.8708
p = 0.5786
0
1
2
GCL
MSS
CA1sp
C
Wild-type
App^NL-F/NL-F
RD
HFD
RD
HFD
75-
50-
37-
4-HNE
45 kDa
50-
37-
β–actin
D
p < 0.0001
***
Relative level of 45-kDa 4-HNE-protein
0
2
4
6
8
10
RD
HFD
RD
HFD
Wild-type
App^NL-F/NL-F

Supplementary Figure S6. A high-fat diet did not induce neurodegeneration or enhance protein modification through lipid peroxidation in wild-type or *App*$^{NL\text{-}F/NL\text{-}F}$ mice. (A) Fluoro-Jade C staining revealed no degenerating neurons in the hippocampus of wild-type and *App*$^{NL\text{-}F/NL\text{-}F}$ mice fed an RD or HFD. Scale bar = 200 μm. (B) The quantitative measurement of the Fluoro-Jade C intensity in three zones of the hippocampus (GCL, MSS, and CA1sp) revealed no significant change in the intensity among all groups. Data are expressed as the mean ± SEM, n = 4, and 4 brain slices per mouse were examined. The results were statistically analyzed by a two-way ANOVA (*p* values are shown). (C) Representative Western blots showing increased levels of 4-hydroxynonenal (4-HNE)-modified proteins in in hippocampus of *App*$^{NL\text{-}F/NL\text{-}F}$ mice fed an HFD. (D) Quantification of 4-HNE-modified proteins in blots using β-actin as a loading control. Intensity of a 45-kDa band with strong anti-4-HNE reactivity was quantified. The bar graph shows the 45-kDa 4-HNE-protein/β-actin ratio relative to RD-fed wild-type mice. Data are expressed as the mean ± SEM, n = 3. The results were statistically analyzed by a two-way ANOVA (*p* value is shown) followed by post-hoc Tukey's HSD test, $^{***}p < 0.001$. *App*$^{NL\text{-}F/NL\text{-}F}$ mice exhibited markedly increased levels of protein modification via lipid peroxidation in the hippocampus compared with wild-type mice, but HFD administration induced no marked changes in either genotype.

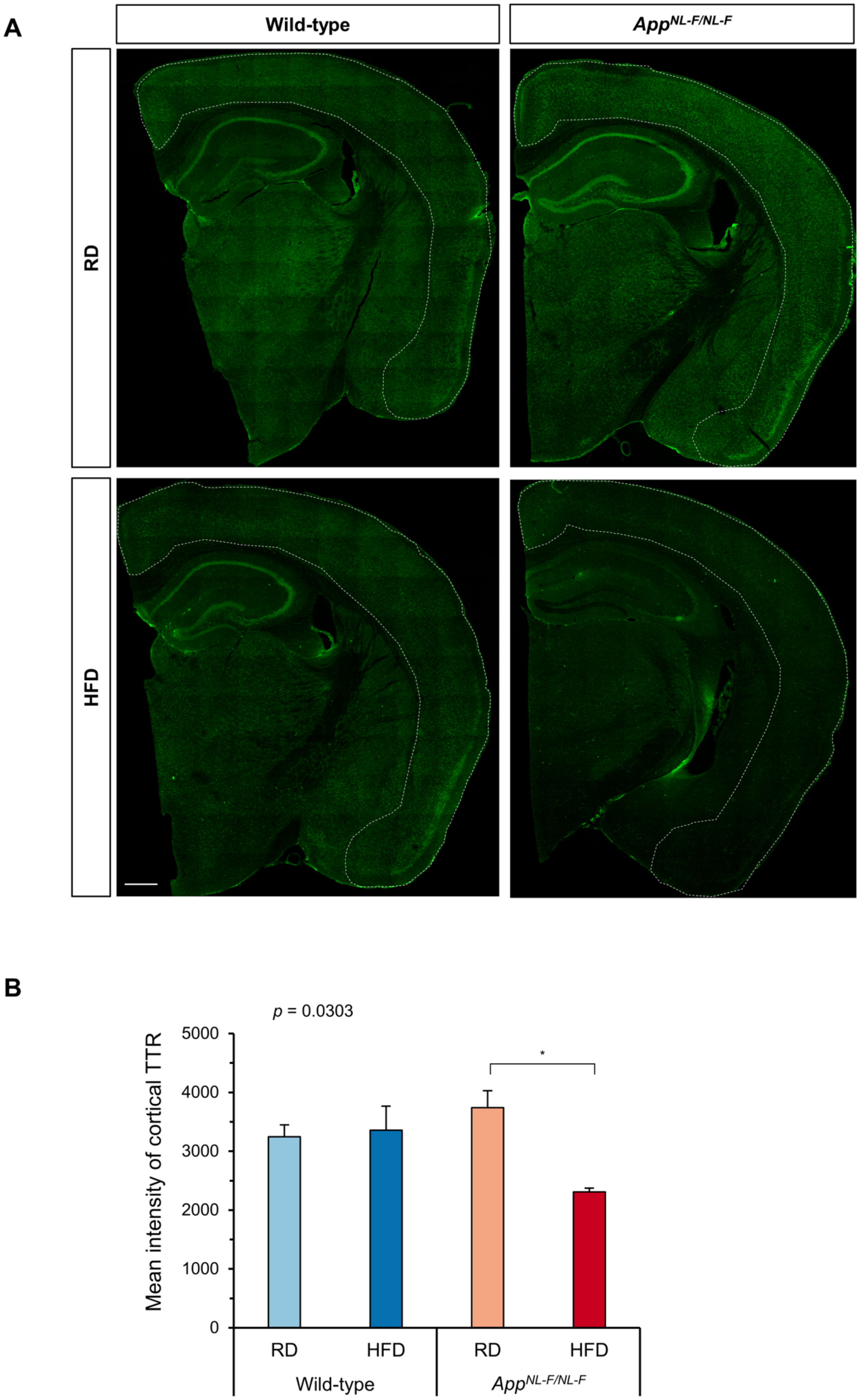
A
Wild-type
$App^{NL-F/NL-F}$
RD
HFD
B
p = 0.0303
Mean intensity of cortical TTR
5000
4000
3000
2000
1000
0
*
RD
HFD
RD
HFD
Wild-type
$App^{NL-F/NL-F}$

Supplementary Figure S7. Quantification of transthyretin expressed in the cortex by immunofluorescence microscopy. (A) Representative immunofluorescence images of transthyretin in the cortex of *App*$^{NL\text{-}F/NL\text{-}F}$ and wild-type mice fed an RD or HFD. The regions of interest for quantification are shown by dotted lines. Scale bar = 500 μm. (B) The quantitative measurement of transthyretin (TTR) in the cortex shows a significantly decreased level in *App*$^{NL\text{-}F/NL\text{-}F}$ mice fed an HFD. Data are expressed as the mean ± SEM. n = 3, and 3 brain slices per mouse were examined. The results were statistically analyzed by a two-way ANOVA (*p* values are shown for each analysis) followed by post-hoc Tukey's HSD test, $^{*}p < 0.05$.

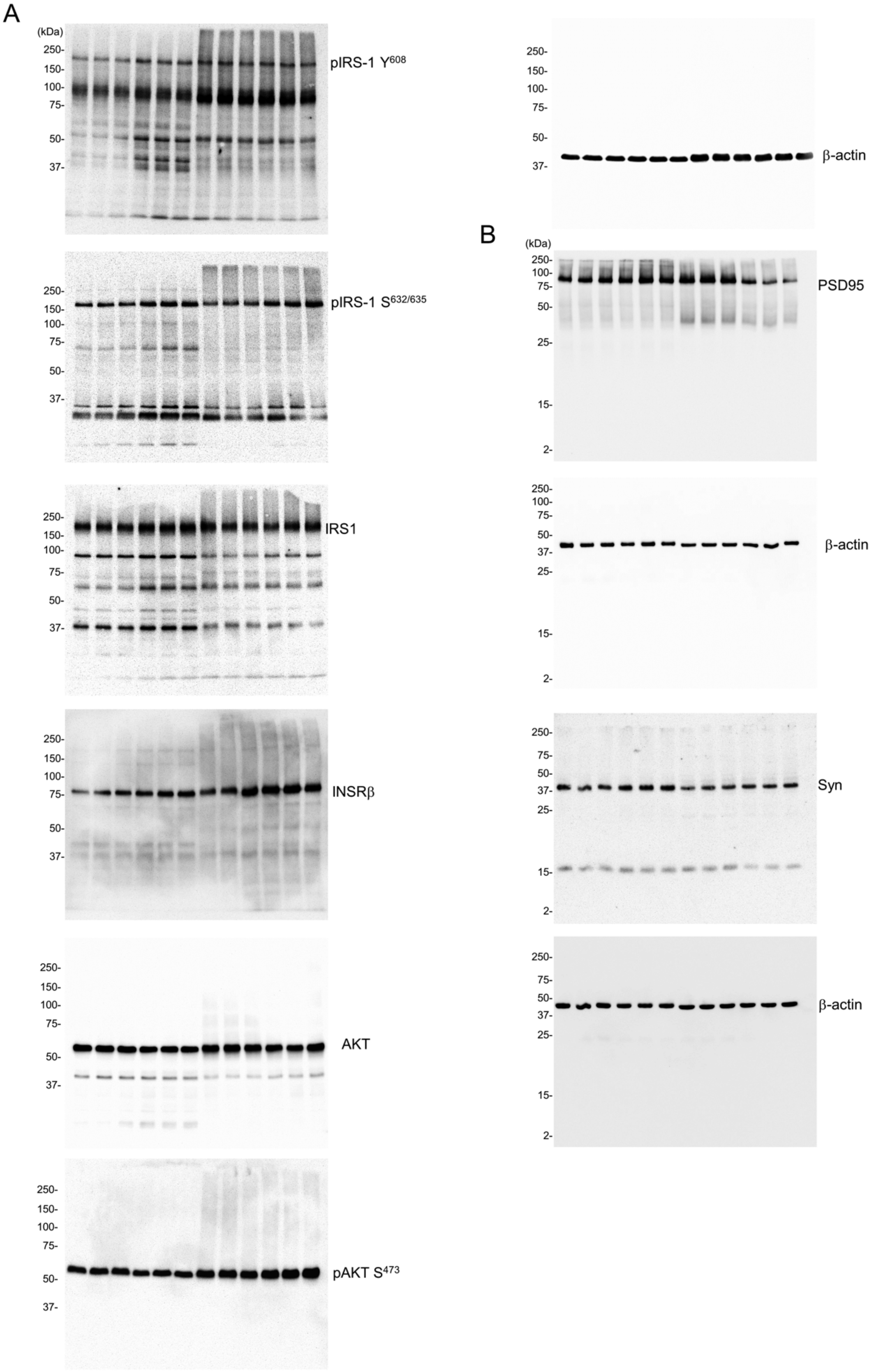

Supplementary Figure S8. Full Western blot images. (A) Uncropped blots for Figure 1C. (B) Uncropped blots for Figure 2D.

A

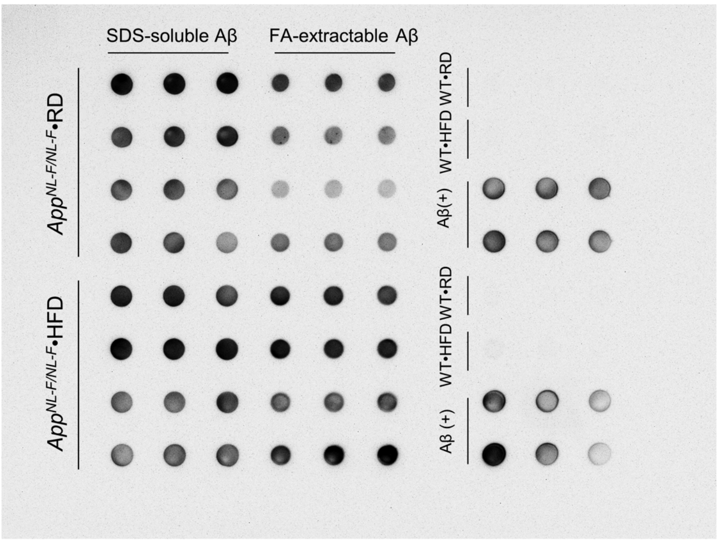

B

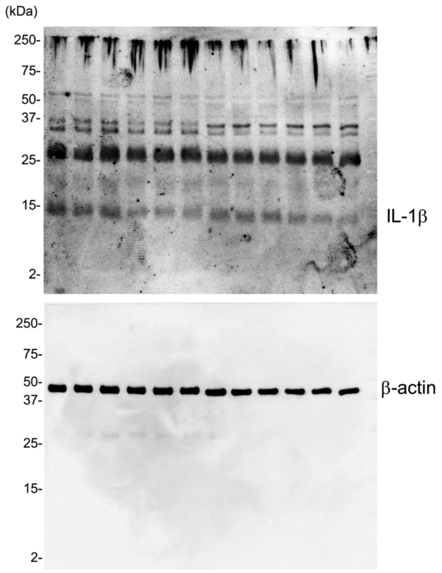

C

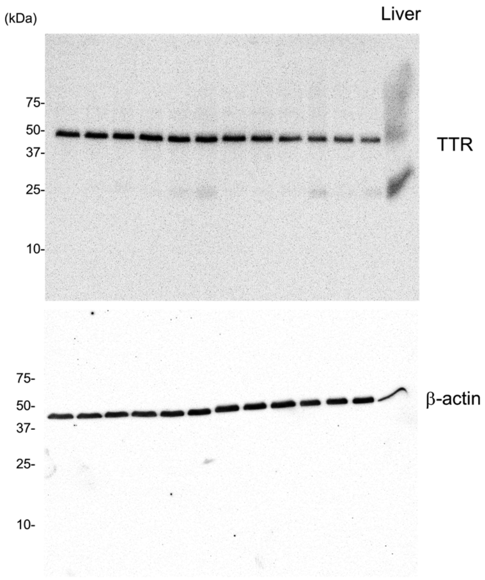

Supplementary Figure S9. Full dot blot and Western blot images. (A) Uncropped blots for Figure 3E. (B) Uncropped blots for Figure 4D. (C) Uncropped blots for Figure 6C.

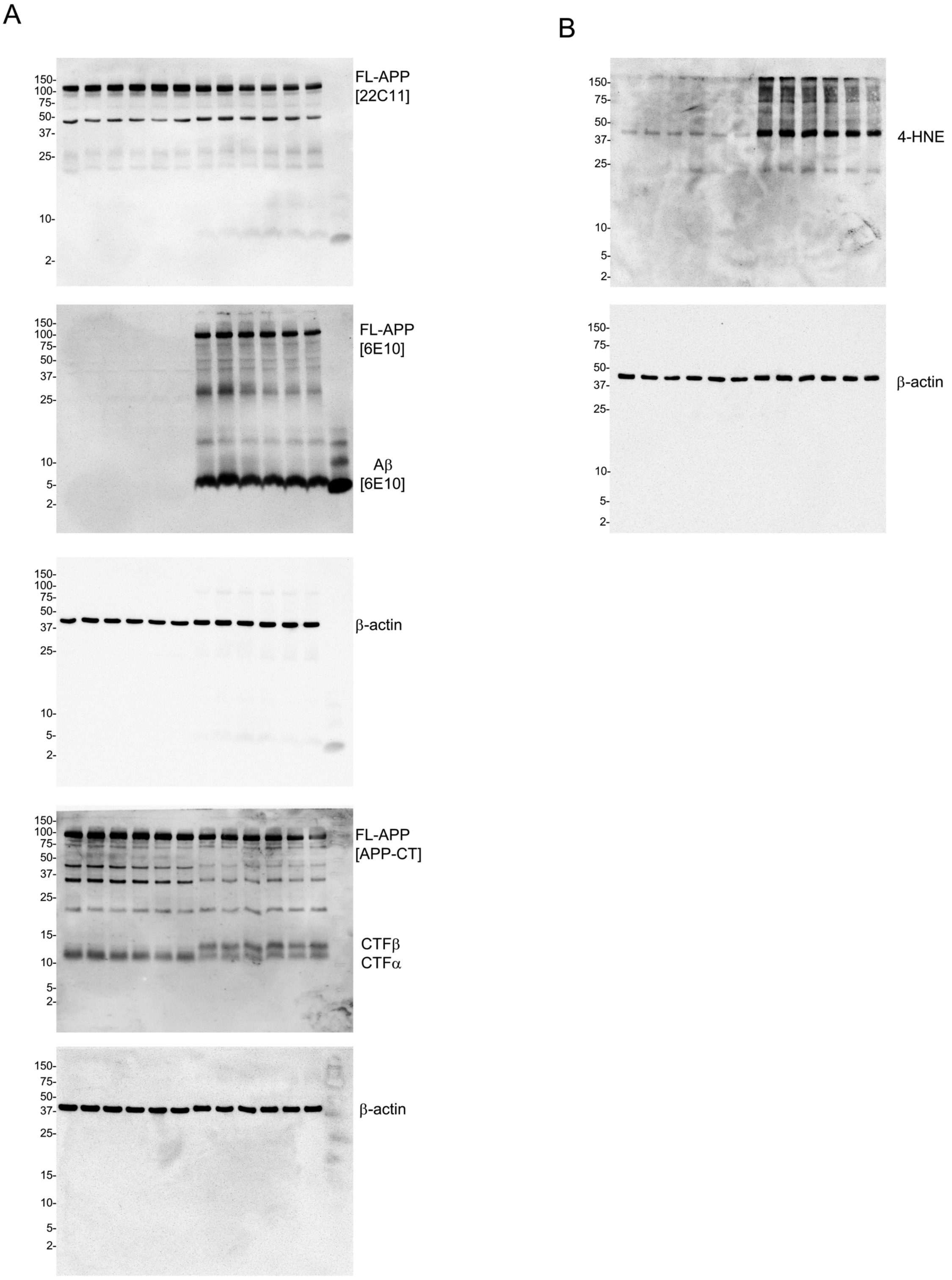

Supplementary Figure S10. Full Western blot images for Supplementary Figures. (A) Uncropped blots for Supplementary Figure S4A. (B) Uncropped blots for Supplementary Figure S6B.
